# A complex interplay of intra- and extracellular factors regulates the outcome of fetal- and adult-derived MLL-rearranged leukemia

**DOI:** 10.1038/s41375-024-02235-5

**Published:** 2024-03-30

**Authors:** Maria Jassinskaja, Sudip Ghosh, Joanna Watral, Mina Davoudi, Melina Claesson Stern, Ugarit Daher, Mohamed Eldeeb, Qinyu Zhang, David Bryder, Jenny Hansson

**Affiliations:** 1 Lund Stem Cell Center, Department of Experimental Medical Science, Lund University, SE-221 84 Lund, Sweden; 2 York Biomedical Research Institute, Department of Biology, University of York, YO10 5DD York, UK; 3 Lund Stem Cell Center, Department of Laboratory Medicine, Lund University, SE-221 84 Lund, Sweden

**Keywords:** Leukaemia, Haematopoiesis, Cell signalling, Cancer microenvironment, Cancer stem cells

## Abstract

Infant and adult *MLL1/KMT2A*-rearranged (MLLr) leukemia represents a disease with a dismal prognosis. Here, we present a functional and proteomic characterization of *in utero*-initiated and adult-onset MLLr leukemia. We reveal that fetal *MLL::ENL*-expressing lymphomyeloid multipotent progenitors (LMPPs) are intrinsically programmed towards a lymphoid fate but give rise to myeloid leukemia in vivo, highlighting a complex interplay of intra- and extracellular factors in determining disease subtype. We characterize early proteomic events of *MLL::ENL*-mediated transformation in fetal and adult blood progenitors and reveal that whereas adult pre-leukemic cells are mainly characterized by retained myeloid features and downregulation of ribosomal and metabolic proteins, expression of *MLL::ENL* in fetal LMPPs leads to enrichment of translation-associated and histone deacetylases signaling proteins, and decreased expression of inflammation and myeloid differentiation proteins. Integrating the proteome of pre-leukemic cells with their secretome and the proteomic composition of the extracellular environment of normal progenitors highlights differential regulation of Igf2 bioavailability, as well as of VLA-4 dimer and its ligandome, upon initiation of fetal- and adult-origin leukemia, with implications for human MLLr leukemia cells’ ability to communicate with their environment through granule proteins. Our study has uncovered opportunities for targeting ontogeny-specific proteomic vulnerabilities in *in utero*-initiated and adult-onset MLLr leukemia.

## Introduction

Acute leukemias with rearrangements of the Mixed lineage leukemia 1 (*MLL1/KMT2A*) gene represent an aggressive class of the disease with a poor prognosis and response to treatment in infants as well as in adults [1–4]. Infant *MLL1/KMT2A*-rearranged (henceforth MLLr) leukemias are thought to be frequently, if not always, initiated already *in utero* [5, 6]. Critically, a marked discrepancy exists in the incidence rate as well as occurrence of different disease phenotypes between infants and adults; MLLr leukemia predominately manifests as acute lymphoblastic leukemia (ALL) in infants, while acute myeloid leukemia (AML) presents at similar frequency as ALL in MLLr leukemia in adults [7]. Although the reasons behind this discrepancy remain elusive, the fact that fetal and adult hematopoiesis differ greatly on both a functional and a molecular level [8–14] together with the studies that have highlighted that the developmental age and lineage potential of the leukemia-initiating cell (LIC) plays a crucial role in both the type and the aggressiveness of the subsequent disease are of high relevance [15–17]. Importantly, in MLLr leukemia, also the developmental stage of the niche in which the disease is propagated has been implicated as an important factor in regulating both the aggressiveness and phenotype of the leukemia [18–21].

In this work, we have performed a comprehensive proteomic and functional characterization of MLLr leukemic transformation in fetal- and adult-origin LICs and linked the ontogenic differences to the proteomic composition of the cells’ respective extracellular environment. We have further interrogated the role of this interplay on the cells’ secretome in mouse as well as human. We delineate ontogenically conserved and developmentally regulated molecular events in *MLL*::Eleven Nineteen Leukemia (*ENL/MLLT1*)-mediated transformation and provide evidence for an age-dependent difference in leukemia cell phenotype mediated by cell-intrinsic as well as niche-associated factors. Our work has identified promising age-tailored novel therapeutic targets to effectively treat acute leukemias driven by *MLL::ENL*.

## Results

### Fetal *MLL::ENL*-expressing LMPPs give rise to progeny with an immature lymphoid immunophenotype in vitro

**Fig. 1:**
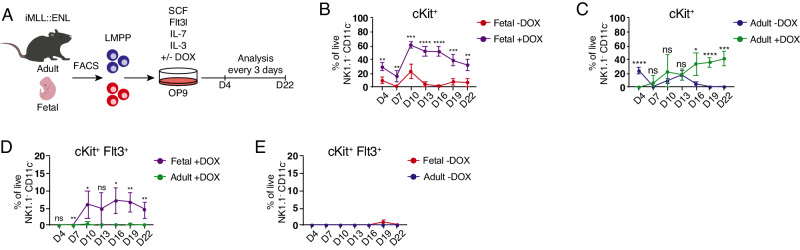
LMPPs expressing *MLL::ENL* show ontogenic differences in lineage output in vitro. **A** Workflow for in vitro differentiation of fetal and adult LMPPs derived from iMLL::ENL mice. Expression of the oncogene was induced by addition of doxycycline (DOX) to the cultures. **B**, **C** Frequency of cKit^+^ cells derived from fetal (**B**) and adult (**C**) *MLL::ENL*-induced (+DOX) and non-induced (−DOX) LMPPs. **D**, **E** Frequency of cKit^+^ Flt3^+^ derived from fetal and adult *MLL::ENL*-expressing (**D**) and normal (**E**) LMPPs. *n* = 4 for all displayed graphs. Error bars represent SD. ****p < 0.0001, ***p < 0.001, **p < 0.01, and *p < 0.05 and ns not significant. See also Fig. S1.

Upon induced expression of the *MLL::ENL* as well as *MLL::AF4* oncogenes, lymphomyeloid hematopoietic progenitor cells represent the most potent LICs among early hematopoietic stem and progenitor cells (HSPCs) [22–24]. Lymphomyeloid multipotent progenitors (LMPPs; Lin^−^ Sca-1^+^ cKit^+^ (LSK) Flt3^high^ CD150^−^) additionally show marked differences in both proteotype and differentiation-associated functions [11] and thus represent highly interesting targets for exploration of the ontogenic effect on their leukemic potential. In humans, the *MLL::ENL* fusion (resulting from chromosomal translocation (11;19)(q23;p13.3)) is most commonly associated with ALL in infants as well as in adults; however, the mutation almost never gives rise to AML in infant children whereas 22% of adults with the mutation present with myeloid disease [7]. To investigate the lineage potential of normal relative to transformed fetal (embryonic day [E] 14.5) and adult (6–10 week old) LMPPs, we performed an in vitro differentiation assay using a mouse model harboring doxycycline (DOX)-inducible expression of *MLL::ENL* (iMLL::ENL mice) [23] (Fig. 1A). We observed retained expression of the leukemia stem cell (LSC)-associated marker cKit [25, 26] already at 4 days after induction of *MLL::ENL* expression and a high production of cKit^+^ progeny from transformed cells in cultures initiated with fetal LMPPs (Fig. 1B). In contrast, the frequency of cKit^+^ cells from adult *MLL::ENL*-expressing LMPPs surpassed the control cells with delay compared to fetal cells, only at day 16 of culture (Fig. 1C).

Myeloid cell output was low from fetal as well as adult *MLL::ENL*-expressing LMPPs (Fig. S1A), whereas B cell output increased in a near-linear fashion during the culture period (Fig. S1B). Of note, while the frequency of CD11b^+^ cells in fetal wells was very low at all assayed time points, up to 50% of adult LMPPs intermittently produced high numbers of myeloid progeny (Fig. S1A), indicating an ontogenic difference in the myeloid potential of *MLL::ENL*-expressing LMPPs, as previously observed for WT LMPPs in a similar culture setting [11] as well as in this experiment (Fig. S1C, D). Interestingly, a population of cKit^+^ Flt3^+^ cells emerged in *MLL::ENL*-induced fetal wells at day 7 of culture (Fig. 1D). Critically, the same population was almost completely absent from adult DOX-treated wells at all investigated time points, and very few, if any, cKit^+^ Flt3^+^ cells were produced by fetal and adult LMPPs in the absence of DOX (Fig. 1D, E). Within the hematopoietic system, co-expression of cKit and Flt3 can be found on LMPPs as well as common lymphoid progenitors (CLPs) [27, 28]. In addition, Flt3 expression has been proposed as a hallmark of ALLs harboring MLL rearrangements [29], indicating that fetal, but not adult, *MLL::ENL*-expressing LMPPs give rise to a lymphoid-like leukemic population in vitro.

Collectively, the results from our in vitro assay show that induction of *MLL::ENL* in fetal and adult LMPP confers LSC properties to these cells and indicate a stronger lymphoid bias in fetal compared to adult *MLL::ENL*-expressing LMPPs.

### Fetal and adult LMPPs expressing *MLL::ENL* give rise to aggressive leukemia in vivo

**Fig. 2:**
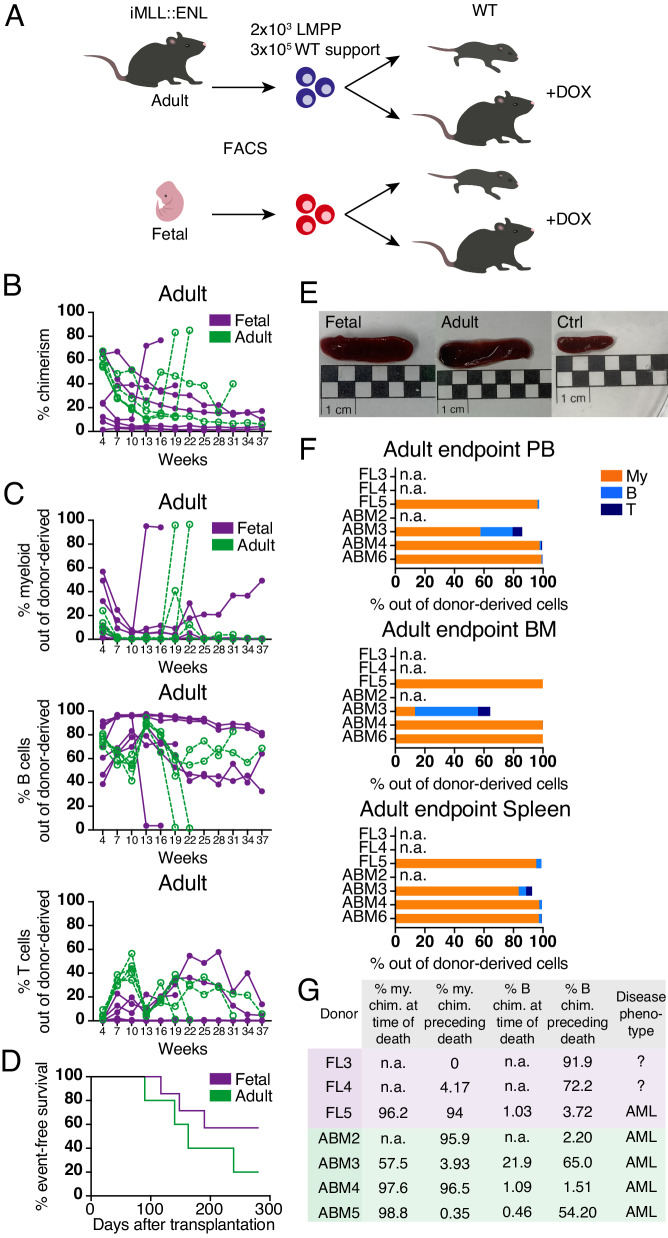
Fetal and adult LMPPs expressing *MLL::ENL* give rise to aggressive leukemia in vivo. **A** Workflow for transplantation of fetal and adult iMLL::ENL LMPPs into WT adult and neonatal recipients. *n* = 4 per group for neonatal recipients and *n* = 7 and 5 for adult recipients of fetal and adult iMLL::ENL LMPPs, respectively. For neonatal recipients, the pregnant females were put on a DOX-containing diet at E18.5, and adult recipients were put on a DOX-containing diet 4 days prior to transplantation. **B** Donor chimerism in peripheral blood of adult recipients transplanted with fetal or adult iMLL::ENL LMPPs. **C** Myeloid, B- and T cell chimerism in adult recipients transplanted with fetal or adult iMLL::ENL LMPPs. **D** Survival of adult recipients transplanted with fetal or adult iMLL::ENL LMPPs. **E** Representative images of spleens harvested from moribund or deceased mice (transplanted with fetal (left) or adult (middle) iMLL::ENL LMPPs) and healthy controls (right). **F** Distribution of myeloid, B- and T cells in peripheral blood (PB), bone marrow (BM), and spleen of leukemic mice at time of death. Donor sample names are listed on the y-axis. FL fetal liver and ABM adult bone marrow. **G** Classification of leukemia subtype in adult recipients transplanted with iMLL::ENL fetal or adult LMPPs. Myeloid and B cell chimerism in PB at time of death and/or in the last assayed timepoint before death. Donor sample names are listed. n.a. refer to recipients that died before the onset of detectable morbidity. FL fetal liver, ABM adult bone marrow, my myeloid, B B cell, chim chimerism. See also Fig. S2.

Next, we performed an in vivo assay of the leukemic potential of fetal and adult LMPPs via transplantation into WT recipients (Figs. 2A and S2A). To account for the effect of the niche developmental stage in which MLLr leukemia is propagated [20], we included adult as well as neonatal (24–48 h old) recipients. Adult cells engrafted poorly in neonatal recipients, with no peripheral blood (PB) chimerism above 0.2% by 37 weeks post-transplantation (Fig. S2B). Chimerism in neonates transplanted with fetal cells remained above 1% in 3 out of 4 recipients throughout the assayed period, and only fetal cells gave rise to multilineage progeny in mice that were neonatally transplanted (Fig. S2B–E). However, none of the neonatal recipients developed leukemia during the experiment.

43 and 80% of adult recipients of fetal and adult cells, respectively, succumbed to disease, with recipients receiving adult cells showing a shorter latency (Fig. 2B–E). One fetal and two adult iMLL::ENL LMPP transplanted adult recipients showed a spike in myeloid chimerism, a dramatic drop in B cell chimerism (Fig. 2C), as well as a sharp increase in white blood cell (WBC) count (Fig. S2F) shortly before death, indicating a highly aggressive acute leukemia. Diseased adult cell recipients showed almost exclusively myeloid chimerism at the time of or shortly preceding death (Fig. 2F, G), confirming that adult LMPPs give rise to AML upon induced expression of *MLL::ENL* in vivo [23, 24]. AML could additionally be confirmed in one recipient of fetal LMPPs (FL5; Fig. 2G). Although the remaining two diseased recipients of fetal cells died before the onset of detectable morbidity, no signs of myeloid disease could be observed in PB collected within three weeks before death, at which point most of the donor-derived cell pool consisted of B cells (Figs. 2G, S2G, H).

Taken together, our results show that both fetal and adult LMPPs can act as potent LICs upon expression of *MLL::ENL* and give rise primarily to AML in vivo. This contrasts our in vitro findings, hence indicating that the adult microenvironment promotes myeloid programs in cells expressing *MLL::ENL*, while the neonatal microenvironment supports the fetal counterpart to a greatly elevated capacity to engraft and sustain multilineage hematopoiesis.

### Protein expression accurately separates fetal and adult LMPPs on ontogenic and disease state

**Fig. 3:**
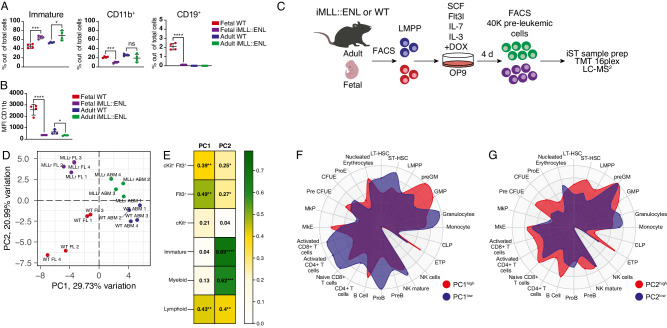
Protein expression accurately separates fetal and adult LMPPs on ontogenic and disease state. **A**, **B** Frequency of immature (CD11b^−^ CD19^−^), CD11b^+^, and CD19^+^ cells (**A**) and CD11b expression on CD11b^+^ cells (**B**) in 4-day cultures of fetal and adult WT and iMLL::ENL LMPPs in the presence and absence of DOX. *n* = 4 for all conditions. **C** Workflow for proteomic analysis of early events of MLL::ENL leukemia initiation in fetal and adult LMPPs. LMPPs were derived from WT and iMLL::ENL mice and expression of the oncogene was induced by addition of DOX to the cultures. After 4 days, 40,000 NK1.1^−^ CD11c^−^ B220^−^ Ly6G^−^ cells were FACS-sorted and subjected to quantitative mass spectrometry. *n* = 4. **D** PCA based on the top 300 most variably expressed proteins in fetal and adult WT- and iMLL::ENL LMPP-derived 4-day cultures. **E** Pearson correlation between principal component (PC) 1 and 2 and cellular composition of proteome samples as seen in Fig. 3A (i.e. % Immature, Myeloid, and Lymphoid), as well as % Flt3^+^, cKit^+^, and cKit^+^Flt3^+^ cells. **F**, **G** Radar plots depicting the association of loadings for PC1 (**F**) and PC2 (**G**) with known transcriptional profiles of murine hematopoietic cell subsets [33]. In broad terms, PC1 represents the effect of age (PC1^low^: fetal, PC1^high^: adult) whereas PC2 represents the effect of oncogene expression (PC2^low^: WT, PC2^high^: MLL::ENL). Error bars represent SD. ****p < 0.0001, ***p < 0.001, **p < 0.01, and *p < 0.05 and ns not significant. See also Fig. S3 and Table S1.

Having established the leukemic potential of fetal and adult *MLL::ENL*-expressing LMPPs, we set out to delineate the molecular features governing the earliest stages of leukemic transformation. Following 4 days of culture in the presence of DOX, a block in differentiation was already evident in fetal and adult iMLL::ENL LMPPs (Fig. 3A, B). We FACS-sorted 40,000 live NK1.1^−^ CD11c^−^ B220^−^ Ly6G^−^ cells per sample from these cultures in four biological replicates and subjected the cells to a quantitative mass spectrometry (MS)-based proteomic workflow adapted for low cell numbers (Fig. 3C) [11, 30–32]. We identified 3585 proteins out of which 2769 were quantified across all four conditions with high reproducibility (Fig. S3A, B and Table S1).

Principal component analysis (PCA) of the 300 most variably expressed proteins showed a clear separation of samples on both ontogenic state and oncogene expression (Fig. 3D). PCA indicated greater separation between fetal WT and *MLL::ENL*-expressing cells than the respective populations in the adult, suggesting more pronounced molecular changes in the fetus compared to the adult upon onset of pre-leukemia, and/or that these molecular changes occur faster in the fetus. Both principal component (PC) 1 and PC2 showed significant correlation with the cellular composition of the assayed samples (Fig. 3E). Intriguingly, PC1, which most clearly separated fetal and adult samples (Fig. 3D), showed a particularly high correlation with the frequency of Flt3^+^ cells in the samples, in line with the lymphoid bias of fetal pre-leukemic cells observed in vitro (Fig. 1D). We extracted the loadings for PC1 and PC2 and examined which hematopoietic cell types these proteins are associated to [33] (Fig. 3F, G). Samples further left in the PCA plot (“PC1^low^”; fetal WT and fetal *MLL::ENL* samples) showed strong association with several different lymphoid cell types, including ProB and T cells (Fig. 3F). PC1^high^ samples (adult WT and adult *MLL::ENL*) on the other hand showed stronger association with myeloid progenitors. PC2^high^ samples, which include fetal as well as adult MLLr cells, showed stronger association with the most immature hematopoietic progenitors than the WT (PC2^low^) samples (Fig. 3G).

Collectively, this data shows that protein expression strongly separates healthy and pre-leukemic cells, as well as fetal and adult cells, as early as four days after induction of oncogene expression. In line with the behavior of healthy LMPPs [11], PCA additionally suggests a stronger retainment of lymphoid features in fetal cells compared to adult upon oncogene expression, whereas the opposite is true for myeloid features.

### Proteome analysis identifies ontogeny-specific and ontogenically conserved features of *MLL::ENL*-mediated transformation

**Fig. 4:**
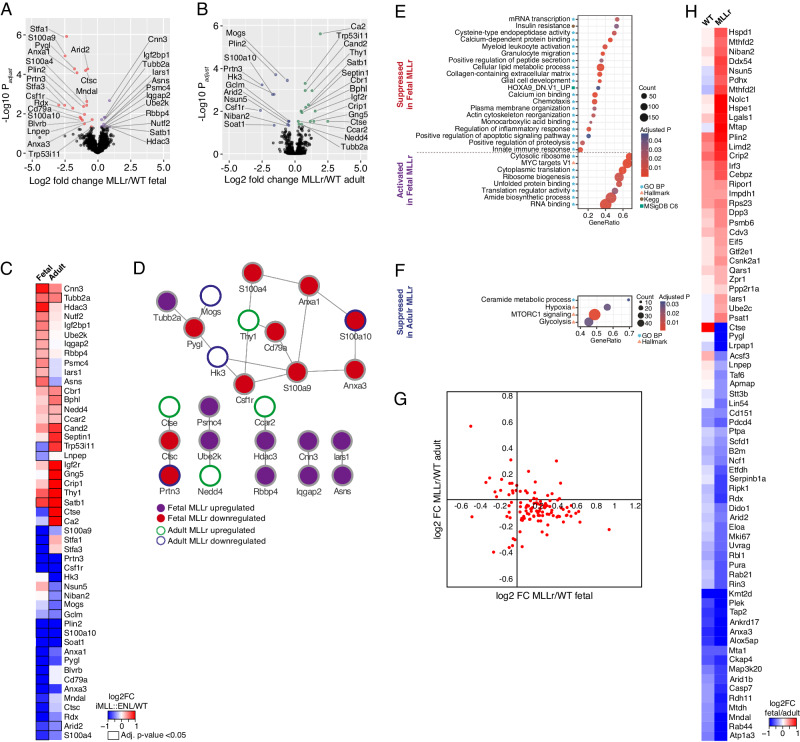
Proteome analysis identifies ontogeny-specific and ontogenically conserved features of *MLL::ENL*-mediated leukemic transformation. **A**, **B** Volcano plots of statistical analysis of proteins differentially expressed between *MLL::ENL*-expressing and WT cells of fetal (**A**) and adult (**B**) origin. Significantly changed proteins (adjusted *p*-value < 0.05) are shown in color. **C** Heatmap depicting average log2 fold change of proteins significantly differentially expressed between pre-leukemic and WT cells in fetus or adult. **D** Protein network of significantly differentially expressed proteins between pre-leukemic and WT cells in fetus or adult. Edges represent known and predicted protein–protein interactions from STRING. Node fill color represents up- or downregulated in fetal MLLr. Node border color depicts up- or downregulated in adult MLLr. **E**, **F** Gene Set Enrichment Analysis (GSEA) in *MLL::ENL* vs WT comparisons in fetus (**E**) and adult (**F**). Gene sets with positive normalized enrichment scores (NES) are termed ‘activated’ and negative NES are termed ‘suppressed’. Count refers to the number of enriched individual proteins and GeneRatio represent (count of enriched proteins)/(gene set size). The symbol next to the GSEA term shows the GSEA database of the respective gene set. No gene set was found statistically significantly activated in adult MLLr. **G** log2 fold change MLLr/WT in fetal and adult for proteins belonging to the 40S and 60S ribosomes. **H** Average log2 fold change of proteins significantly differentially expressed between fetal and adult MLLr cells, and not significantly differentially expressed between fetal and adult WT cells. See also Fig. S4 and Table S2.

Statistical analysis identified 31 and 25 proteins as differentially expressed (adjusted *p*-value < 0.05) between WT and *MLL::ENL*-expressing cells in fetus and adult, respectively (Figs. 4A–C and S4A). STRING analysis [34] of significantly changed proteins revealed several of these to be interaction partners acting within the same network (Fig. 4D). Among proteins significantly downregulated upon oncogene expression, 5 proteins were shared between fetus and adult: Arid2, Csf1r, Plin2, S100a10, and Prtn3, with several others (e.g. S100a9, Anxa1, Anxa3, and Soat1) showing concordant average expression changes but only reaching statistical significance in one of the two comparisons (Figs. 4C and S4A). Many of these proteins (Arid2, Csf1r, Prtn3, Anxa1, Anxa3, and S100a9) are associated with terminal differentiation [35, 36], and the decrease in their expression is thus in line with the rapid differentiation block induced by *MLL::ENL* (Fig. 3A, B). Soat1 and Plin2 highlight lipid storage as another shared feature downregulated upon *MLL::ENL* induction. Shared proteins upregulated in the pre-leukemic samples included Satb1, Tubb2a, Cnn3, Nedd4, and Cand2 (Fig. 4C). Satb1 has previously been shown to be associated with heightened hematopoietic stem cell (HSC) self-renewal [37], whereas the role of Tubb2a, Cnn3, Nedd4, and Cand2 in hematopoiesis and/or leukemia is poorly described. Mapping protein expression to genes previously identified as differentially expressed between normal and pre-leukemic adult pre-granulocytes-monocyte (pGM) progenitor cells [23] showed an overall agreement between adult upregulated protein and mRNA, while Ctse transcript was downregulated and the corresponding protein upregulated (Fig. S4B).

Several proteins only showed expression changes in one of the two comparisons, or even opposing changes upon leukemia initiation in fetal versus adult cells. Particularly interesting examples are components of the histone deacetylase (HDAC) signaling pathway – Hdac3 and Rbbp4 – which were upregulated in pre-leukemic relative to control cells in fetal but not in adult cells (Fig. 4C). These proteins may contribute to differential sensitivity of infant and adult leukemia to HDAC inhibitors, which have recently emerged as a promising anti-cancer therapy for MLLr leukemia specifically [38–40]. A protein that was strongly upregulated exclusively in adult pre-leukemic cells was Igf2r, which is classified as a growth inhibitor and has also been proposed as a target for tumor control [41]. The opposing expression pattern of the p53 target Trp53i11, with elevated expression in adult cells while reduced in fetal cells upon leukemic transformation, indicates that fetal cells more rapidly suppress apoptotic pathways following mutation acquisition.

Gene set enrichment analysis (GSEA) showed strongly disparate features of leukemic transformation in fetal and adult cells (Fig. 4E, F, and Table S2). Upon expression of *MLL::ENL*, fetal LMPPs showed an upregulation of proteins associated with translation, whereas proteins downregulated upon transformation in adult cells showed enrichment for mTORC signaling, indicating a decrease in translational activity in adult pre-leukemic relative to healthy cells. The opposing changes in expression of translational proteins upon *MLL::ENL* expression, including most proteins of the 40S and 60S ribosomes (Fig. 4G), is particularly intriguing considering that a high versus low translation rate is one of the key differences between normal fetal and adult HSPCs [42]. Since ribosomal genes are enriched in primary cells immortalized by *MLL::ENL* [43], the anticorrelating behavior of associated proteins observed here may indicate that fetal-origin LMPPs take on leukemic features more rapidly than their adult counterpart upon induced expression of *MLL::ENL*.

Proteins significantly downregulated in pre-leukemic relative to normal fetal cells were enriched mainly for gene sets associated with myeloid differentiation and inflammation (Fig. 4E), again highlighting a broad loss of myeloid features in these cells upon expression of *MLL::ENL* as previously discussed (Figs. 1D, 3A, B). Critically, this was not the case for adult pre-leukemic cells, which showed downregulation of proteins associated with glycolysis and hypoxia (in addition to mTORC signaling; Fig. 4F), indicating that metabolic rewiring and adaption to changing oxygen levels are features unique to early leukemogenesis in the adult.

Next, we performed statistical analysis of fetal versus adult cells in WT and MLLr samples and overlayed significantly changed proteins (Figs. 4H, S4C, D, and Table S1). 45 proteins that showed differential expression between fetal and adult WT cells maintained similar expression differences upon oncogene expression (Fig. S4C). Interestingly, this set of proteins contained B cell-associated proteins CD79a and Mzb1 that were elevated in fetal cells, and myeloid proteins Mpo and Ctsg that were higher expressed in adult cells. This again highlights that lineage-associated differences that are present in normal fetal and adult cells are retained upon expression of *MLL::ENL*. The B cell receptor (BCR) component CD79a additionally showed significant downregulation in fetal pre-leukemic cells while remaining unchanged in adult pre-leukemic cells (Fig. 4C). The drop in expression upon induction of *MLL::ENL* in fetal LMPPs is in line with the differentiation block induced by the fusion oncogene (Fig. 3A, B). Aberrant pre-BCR and BCR signaling play a central role in B cell neoplasia, with enhanced positive signaling of the pre-BCR promoting B-ALL [44], and upregulation of CD79a has been proposed to increase the risk for infiltration of the central nervous system in pediatric B-ALL [45]. Analysis of CD79a surface expression on cultured fetal and adult iMLL::ENL LMPPs showed a higher presence of CD79a^+^ cells in fetal compared to adult pre-leukemic samples, and a decrease in CD79a surface expression on adult pre-leukemic relative to uninduced cells (Fig. S4E, F). Together with our proteome data, this points towards dysregulation of signaling via this receptor as a feature associated with *MLL::ENL*-driven transformation, particularly in fetal cells.

Cross-comparison between proteins differentially expressed between fetal and adult, and WT and MLLr, cells revealed a multitude of proteins that were expressed at similar levels between fetal and adult WT cells but showed differential expression when comparing fetal and adult MLLr cells due to changes in their levels upon induction of the oncogene (Fig. 4H). This included several members of the protein folding machinery – specifically, Hspe, Hspd1, and Cebpz. While largely unchanged in adult normal versus pre-leukemic cells, these proteins were upregulated in fetal cells upon induction of *MLL::ENL* (Table S1), leading to differential expression between fetal and adult MLLr cells (Fig. 4H). Upregulation of heat shock proteins (HSPs), in particular Hspd1, has previously been identified as a poor prognostic factor in AML [46]. Further, we found that fetal relative to adult MLLr cells showed elevated expression of Mthfd2l and Mthfd2, which were previously shown to be the most strongly differentially expressed metabolic enzymes in *MLL::AF9* AML samples compared to control cells, and whose knockdown improves survival in a mouse model of the disease [47]. Similarly, the protein Mtap showed differential expression between fetal and adult MLLr cells due to a drop in expression in adult cells upon induction of *MLL::ENL*. Low expression of this phosphorylase has previously been shown to increase the sensitivity of T-ALL cells to purine synthesis inhibition or methionine starvation [48]. Our data thus suggests ontogenic differences in the sensitivity of *MLL::ENL*-driven leukemia to inhibition of pathways under the control of Mthfd2 and Mtap, highlighting potential opportunities for age-tailored therapeutic approaches.

### The proteomic composition of the extracellular environment of fetal and adult HSPCs displays significant differences

**Fig. 5:**
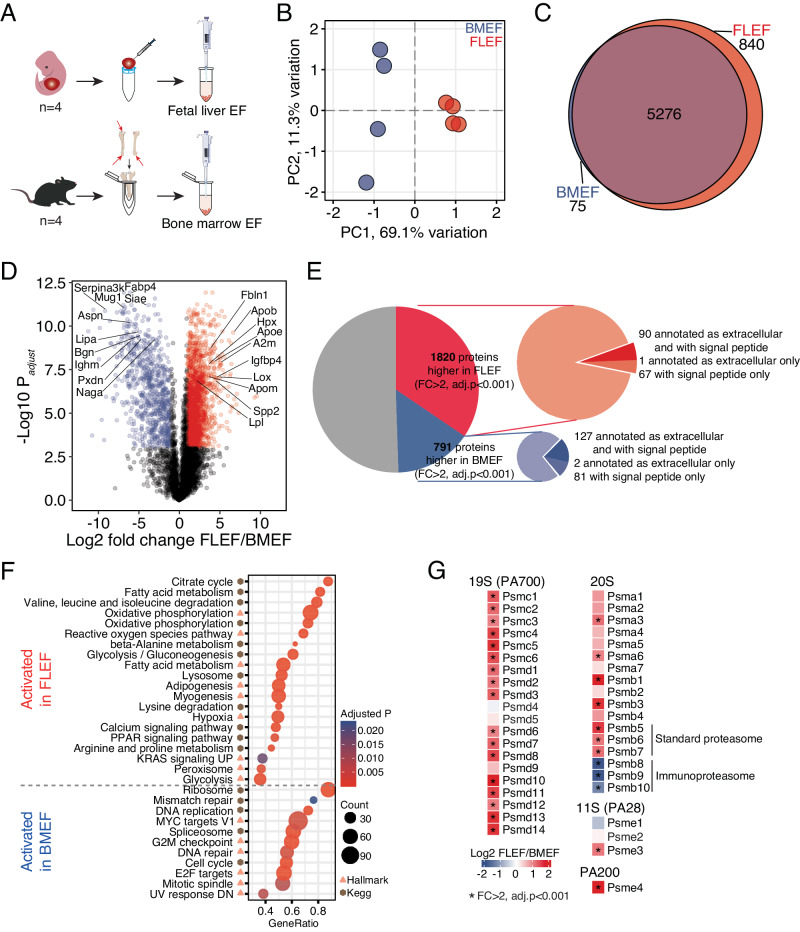
The proteomic composition of the extracellular environment of fetal and adult HSPCs displays significant differences. **A** Workflow for the extraction and processing of fetal liver and bone marrow extracellular fluid (EF) for proteomic analysis. *n* = 4. **B** PCA for the comparison of FLEF and BMEF using all quantified proteins in MSStats. **C** Venn diagram showing overlap between proteins identified in FLEF and BMEF. **D** Statistical analysis of proteins differentially expressed between FLEF and BMEF. Significantly changed proteins (adjusted *p*-value < 0.001, fold change >2) are shown in color and top 10 significant proteins with ‘extracellular’ annotation in FLEF or BMEF are marked. **E** Proportion of significantly changed proteins (adjusted *p*-value < 0.001, fold change >2) out of all quantified proteins in FLEF and BMEF, and proportion of proteins annotated as extracellular and with signal peptides (in dark color). **F** GSEA in FLEF vs BMEF comparisons. Gene sets with positive and negative NES are termed activated in FLEF and BMEF, respectively. Count refers to the number of enriched individual proteins and GeneRatio represent (count of enriched proteins)/(gene set size). The symbol next to the GSEA term shows the GSEA database of the respective gene set. **G** Expression differences between BMEF and FLEF for proteasome 19S, 20S, 11S, and PA200 subunit components. Significantly changed proteins (adjusted *p*-value < 0.001, fold change >2) are marked with star. FC fold change. See also Fig. S5 and Tables S3 and S4.

Our in vitro and in vivo assays highlighted that extracellular factors play a role in influencing the behavior of the cells upon leukemic transformation (Figs. 1 and 2). We therefore sought to determine the proteomic composition of the extracellular milieu where fetal and adult LICs reside, i.e. the extracellular fluid (EF) extracted from the FL and adult bones of WT animals (Figs. 5A and S5A). Low between-tissue correlation (Fig. S5B, C) together with a clear separation in principal component space (Fig. 5B) suggest a difference in complexity between these two extracellular compartments. Although the number of identified proteins showed a significant overlap between BMEF and FLEF, with 5276 proteins identified in both compartments, an additional 840 proteins were uniquely identified in FLEF (Fig. 5C), and 2611 proteins showed differential presence in FLEF and BMEF (fold change >2, adjusted *p*-value < 0.001; Fig. 5D and Table S3). Amongst these, similar to previous EF reports [49], we found 5% (91) FLEF-enriched and 16% (129) BMEF-enriched proteins known to be secreted or annotated as extracellular (Figs. 5E and S5D). The remaining proteins may be secreted through non-canonical secretion pathways such as via extracellular vesicles, and may also represent a certain amount of intracellular leakage.

Among the top 10 extracellular proteins at higher levels in FLEF (Figs. 5D and S5E), we found three apolipoproteins (Apob, Apoe, and Apom). Apolipoproteins are major regulators of cholesterol metabolism and the protein constituents of lipoproteins. Apoe has additionally been associated with regulating HSPC proliferation, myeloid cell expansion, and anti-tumor immunity [50, 51]. In addition to lipid metabolism, GSEA showed strong enrichment for various metabolic processes in FLEF (Fig. 5F and Table S4). This included citrate cycle, oxidative phosphorylation, and amino acid degradation, indicating a broad metabolic signaling active in the liver already during embryonic development. On the other hand, cluster-wise enrichment analysis showed that proteins uniquely and highly abundant in BMEF associate with fatty acid biosynthetic process as well as oxidative phosphorylation (Fig. S5F). For example, four members of NADH dehydrogenase (Ndufa2, Ndufa7, Ndufs5, and Ndufv3) were all uniquely present in the BMEF (Table S3), indicating specific extracellular function for these metabolic enzymes in BMEF and possible unique regulation of the leukemia microenvironment of adults, as suggested for other cancers [52].

We found both Fibulin-1 and Fibronectin (Fbln1 and Fn1, respectively) to be more abundant in FLEF compared to BMEF (Figs. 5D and S5E). Fbln1 is an extracellular matrix (ECM) protein known to be secreted by osteoblasts in the BM HSC niche and to interfere with human CD34^+^ cells’ adhesion to Fn1, and inhibit their proliferation [53]. The higher presence of both Fbln1 and Fn1 in FLEF thus suggests an enhanced impact of their interaction in FLEF compared to BMEF. In addition, we found Lysyl oxidase (Lox), a key protein for ECM organization and a novel therapeutic target for primary myelofibrosis [54], to be more abundant in the FLEF relative to BMEF (Figs. 5D and S5E).

Among processes augmented in BMEF relative to FLEF, we found an enrichment mainly for DNA replication and damage repair pathways (Fig. 5F). We identified several proteins associated with inflammatory response to be enriched in BMEF relative to FLEF (Bgn, Lipa, and Hpx; Figs. 5D and S5E). Differential expression of inflammatory proteins is one of the main distinguishing features between fetal and adult HSPCs [10, 11] and our intracellular proteome data showed even further suppression of these signatures in fetal LMPPs upon expression of *MLL::ENL* (Fig. 4E). Our EF findings suggest that the low inflammatory activity observed in normal and pre-leukemic fetal HSPCs additionally extends to the extracellular environment within the FL. Along the same lines, we found that components of the immunoproteasome (Psmb8, Psmb9, Psmb10) are present at lower levels in FLEF compared to BMEF (Fig. 5G), suggesting that their differential extracellular presence [55–57] affect leukemia burden [58, 59] and may render them sensitive to blockage of proteasome-mediated protein degradation [60].

### Secretome and ligand-receptor networks uncover fetal and adult-specific interplay of intra- and extracellular factors

**Fig. 6:**
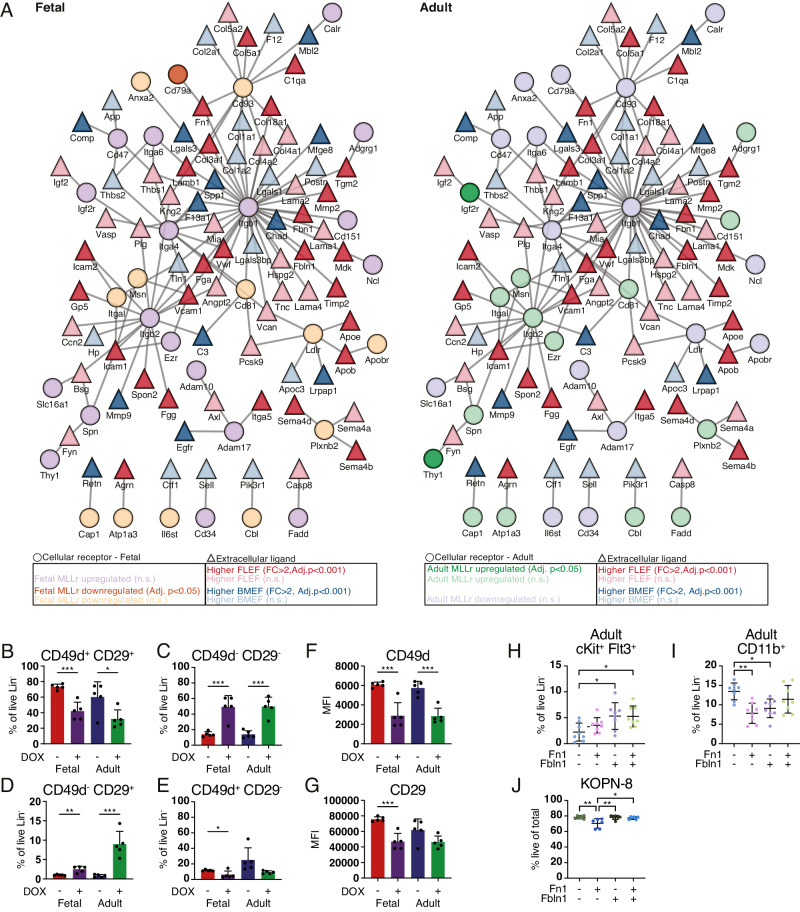
Ontogeny-specific ligand-receptor networks of normal and malignant hematopoiesis. **A** Protein network of extracellular ligands (triangles) of the proteomic comparison of FLEF and BMEF, and cell surface receptors (circles) of the cellular proteomic comparisons of WT and MLLr of fetus (left) and adult (right). Edges represent known and predicted receptor-ligand interactions [61, 62]. FC = fold change, n.s. not significant. **B**–**E** Proportion of cells expressing CD49d and/or CD29 following induction of *MLL::ENL* in fetal and adult LMPPs ex vivo for 4 days. *n* = 5. **F**, **G** Expression level of CD49d (**F**) and CD29 (**G**) following induction of *MLL::ENL* in fetal and adult LMPPs ex vivo for 4 days. n = 5. **H**, **I** Frequency of cKit^+^Flt3^+^ (**H**) and CD11b^+^ (**I**) cells in cultures of adult *MLL::ENL*-expressing cells treated with Fn1 and/or Fbln1 for 4 days. *n* = 8. **J** Viability of KOPN-8 cells following treatment with Fn1 and/or Fbln1 for 2 days. *n* = 6. Error bars represent SD. ****p < 0.0001, ***p < 0.001, **p < 0.01, and *p < 0.05. See also Figs. S6, S7, and S8, and Table S5.

To interpret possibilities of the FL and adult BM microenvironment to influence the onset and initial stages of MLLr leukemia, we interrogated the proteomic data from pre-leukemic cells for cell surface receptors and their corresponding ligands in the EF proteome [61, 62] (Fig. 6A and Table S5). We also characterized homeostatic interactions by mapping receptors from our previously published fetal and adult WT HSPC proteome [10] (Fig. S6A, B). A set of cellular integrins showed prominent differences in their ligandome between FLEF and BMEF. Particularly, 18 of the ligands of Itgb1 (CD29) and four of the ligands of Itga4 (CD49d) showed differential abundance between FLEF and BMEF (Fig. S6B). On leukemic cells, the dimer of these integrins (also known as VLA-4) interacts with the stromal ligands Vcam1 and Fn1 to support attachment to the bone marrow niche [63, 64]. We found both Vcam1 and, as previously highlighted, Fn1 and its antagonist Fbln1, to be higher abundant in FLEF relative to BMEF (Fig. 6A), suggesting a stronger impact of VLA-4/Vcam1 and VLA-4/Fn1 interactions in fetal-origin leukemia. We investigated potential cell surface oncogene-driven changes to the VLA-4 dimer and observed significant, ontogeny-specific changes upon induction of *MLL::ENL* expression (Fig. 6B–G). Upon expression of the oncogene in fetal as well as adult cells, the frequency of double-positive cells was diminished (Fig. 6B), while the frequency of double-negatives increased significantly (Fig. 6C). The frequency of CD29 (Itgb1) single-positive cells also increased for both fetal and adult *MLL::ENL* cells, and this was especially prominent in adult-derived cultures (Fig. 6D). In fetal pre-leukemic cultures, we additionally observed a decrease in CD49d (Itga4) single-positive cells (Fig. 6E). Interestingly, the addition of Fn1 and/or Fbln1 to fetal pre-leukemic cultures reversed the pre-leukemic increase in frequency of double-negative cells (Fig. S7). This further corroborates that the higher presence of Fn1 and Fbln1 in the microenvironment of fetal LICs (Fig. 6A) plays a direct role in influencing their interaction with VLA-4 on the cells. On the other hand, elevating the presence of Fn1 and Fbln1 around adult pre-leukemic cells seems to influence their behavior in terms of directing them towards a more lymphoid phenotype (Fig. 6H, I). While the addition of Fbln1 to the adult cultures increased their lymphoid bias, the addition of Fn1 strongly suppressed their myeloid bias. In addition, out of two infant and two adult MLLr leukemia cell lines assayed in the presence of Fn1 and/or Fbln1 (Fig. S8), only the infant *MLL::ENL* ALL cell line KOPN-8 showed a decrease in viability, which was attributed to Fn1 (Figs. 6J and S8B). While the two adult leukemic cell lines showed robust double-positive VLA-4 dimer expression regardless of Fn1 and Fbln1 presence, the infant cells indicated mild alterations to the dimer with Fbln1 (KOPN-8, Fig. S8G) and Fn1 (THP-1, Fig. S8H).

**Fig. 7:**
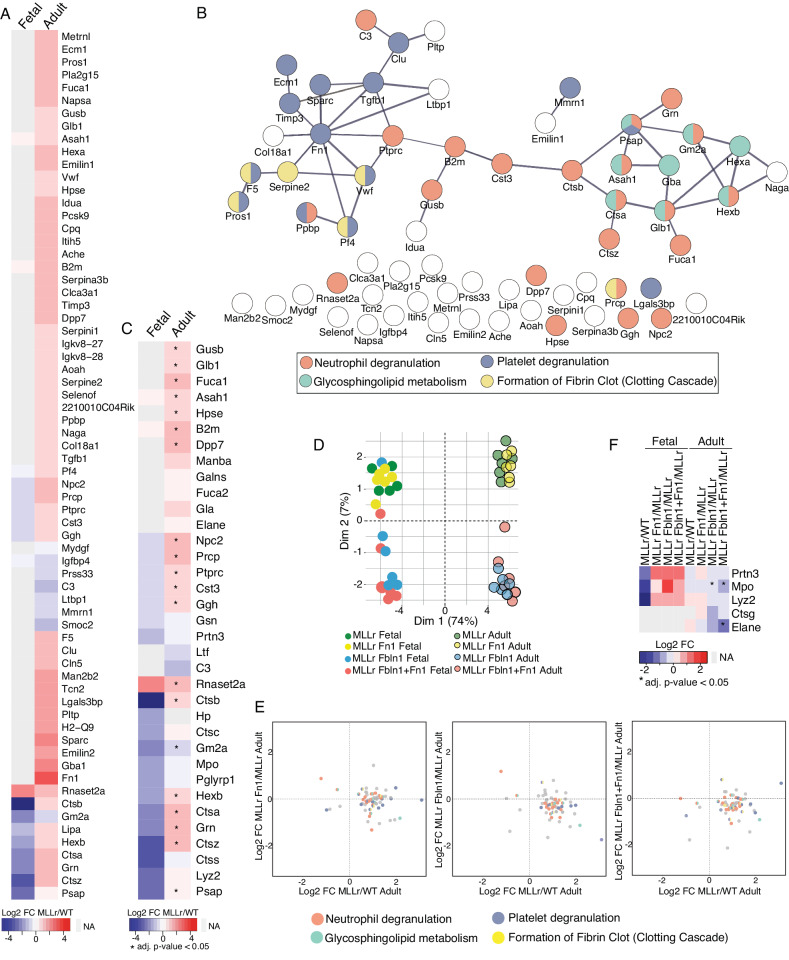
Fetal and adult LMPPs show distinct protein secretion patterns in response to Fn1 and Fbln1 treatment during MLLr leukemia initiation. **A** Heatmap showing average log2 fold change of significantly changed (adjusted *p*-value < 0.05) proteins with ‘extracellular’ annotation in adults but not in fetal between the secretome of pre-leukemic and WT cells. FC = fold change. **B** Protein network of significantly differentially secreted (adjusted *p*-value < 0.05) ‘extracellular’ proteins between the secretome of pre-leukemic and WT cells in adults. Edges represent known and predicted protein–protein interactions from STRING. Node fill colors indicate pathway assignments from Reactome database. **C** Heatmap showing average log2 fold change of ‘extracellular’ proteins involved in the ‘neutrophil degranulation’ pathway (from Reactome) between the secretome of MLLr and WT fetal and adult cells. Significantly changed proteins (adjusted *p*-value < 0.05) are marked with star. **D** PCA for the significantly changed (adjusted *p*-value < 0.05 in at least one group) ‘extracellular’ proteins in the secretome of pre-leukemic fetal and adult cells treated with/without Fn1, Fbln1, and Fbln1 + Fn1. **E** Scatterplots showing the average log2 fold secretion difference between MLLr and WT cells, and between untreated MLLr cells and MLLr cells treated with Fn1, Fbln1, or Fbln1 + Fn1 in adult. The color code corresponds to the involvement of the proteins in the Reactome pathway. **F** Average log2 fold change for the ‘neutrophil primary granule’ proteins in the secretome between MLLr and WT cells, and between untreated MLLr cells and MLLr cells treated with Fn1, Fbln1, or Fbln1 + Fn1 in fetal and adult. adj. adjusted, FC fold change. *n* = 6 for all conditions, except FL WT (*n* = 5) and FL MLLr (*n* = 5). See also Fig. S9 and Table S6.

To investigate the pre-leukemic cells’ potential to influence and communicate with their microenvironment, and what impact ontogeny-specific extracellular factors Fn1 and Fbln1 may have in this communication, we designed a secretome analysis using an albumin-free polyvinyl alcohol (PVA) culture system [65] (Fig. S9A). Intriguingly, fetal and adult pre-leukemic cells showed marked differences in their protein secretion profiles. While adult cells increased their secretion upon *MLL::ENL* induction, fetal pre-leukemic cells instead suppressed secretion (Figs. 7A and S9C), suggesting that adult pre-leukemic cells more actively influence their microenvironment and engage in communication with surrounding cells. Although not statistically significant, amongst the strongest suppressed proteins from fetal cells were several cathepsins (Ctsa, Ctsb, and Ctsz; Fig. 7A). From the adult cells, the strongest induced secretion was found for Fn1, and the majority of statistically significant changes involved proteins related to neutrophil and platelet degranulation pathways (Fig. 7B, C), again highlighting a retention of myeloid features upon induced expression of the oncogene.

When we added Fn1 and/or Fbln1 to pre-leukemic cultures, the secretome profiles distinctly separated the Fn1- and Fbln1- treated groups in PCA (Figs. 7D and S9D), suggesting that Fbln1 has stronger impact than Fn1 on both fetal and adult pre-leukemic secretion. Importantly, Fbln1 supplement to adult pre-leukemic cultures led to a generally suppressed secretion of the proteins that were induced by the expression of *MLL::ENL* (Figs. 7E and S9E). In adults, 6 proteins involved in neutrophil degranulation (Gusb, Ctsb, Rnaset2a, Cst3, Ctsz, Npc2) that showed induced secretion upon oncogene expression showed significant suppression of secretion with the dual Fn1 and Fbln1 treatment (Figs. 7E and S9E). Interestingly, while the overall pattern of differential secretion of neutrophil degranulation proteins from fetal pre-leukemic cells was not evident, a group of neutrophil primary granule proteins showed an interesting reversed pattern of secretion upon the Fn1 and Fbln1 treatment (Fig. 7F). Collectively, our data highlight distinct alterations to granule protein secretion from fetal and adult pre-leukemic cells.

### Integrated proteomic analyses highlight differential modulation of IGF-signaling with impact on leukemic protein secretion

**Fig. 8:**
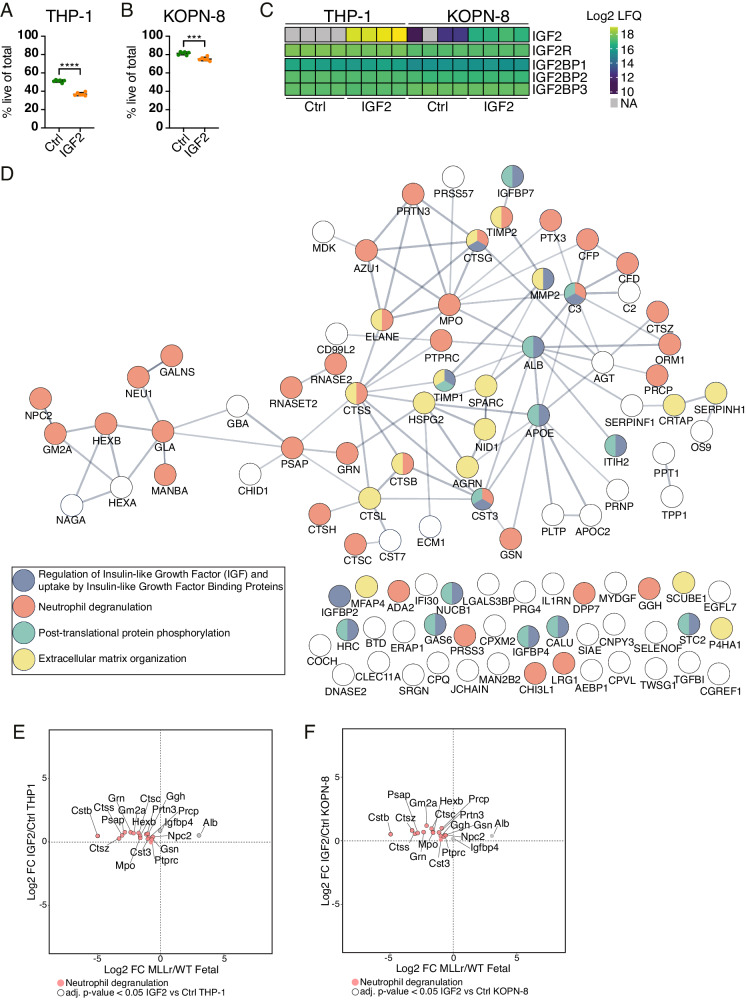
Integrated secretome and phospho-proteome analysis highlight differential modulation of IGF-signaling with impact on leukemic protein secretion. **A**, **B** Viability of THP-1 (**A**) and KOPN-8 (**B**) cells following treatment with IGF2 for 4 days. *n* = 6. Error bars represent SD. ****p < 0.0001, ***p < 0.001. **C** Log2 protein abundance of IGF2, IGF2 receptor (IGF2R), and IGF2 binding proteins (IGF2BP1, IGF2BP2, and IGF2BP3) in THP-1 and KOPN-8 cell lines treated with/without IGF2. LFQ label-free quantification. *n* = 4. **D** Protein network of commonly induced ‘extracellular’ proteins in the secretome of THP-1 and KOPN-8 treated with IGF2. Edges represent known and predicted protein–protein interactions from STRING and node color indicates the Reactome pathway assignments. **E** Scatterplot showing the average relative expression difference in the secretome of mouse MLLr/WT fetal LMPPs (x-axis) and in the secretome of THP-1 cells treated with/without IGF2 (y-axis). Red color mark proteins involved in the neutrophil degranulation pathway and the bold borders represent proteins significantly changed (adjusted p-value < 0.05) between IGF2-treated and control THP-1 cells. Mouse and human proteins were mapped by their gene names. **F** Scatterplot showing the average relative expression difference in the secretome of mouse MLLr/WT fetal LMPPs (x-axis) and in the secretome of KOPN-8 cells treated with/without IGF2 (y-axis). Red color mark proteins involved in the neutrophil degranulation pathway and the bold borders represent proteins significantly changed (adjusted *p*-value < 0.05) between IGF2-treated and control KOPN-8 cells. Mouse and human proteins were mapped by their gene names. See also Figs. S10 and S11 and Tables S7 and S8.

Our intracellular and EF analyses revealed variations in the points of regulation of the insulin-like growth factor (IGF) pathway across ontogeny as well as in MLLr leukemia. We found the receptor for Igf2 (Igf2r) to be significantly upregulated upon *MLL::ENL* expression uniquely in adult LMPPs (Fig. 6A). Of note, the higher expression of Igf2r in fetal cells compared to adult cells in homeostatic conditions is retained in pre-leukemic conditions (Fig. S4D). A higher cell surface expression of Igf2r can be expected to result in decreased IGF-signaling within the cells through Igf1r and insulin receptor [66]. In the extracellular environment, the FLEF showed higher expression of Igfbp4 and Igfbp5 than BMEF, which may contribute to prolonged half-life of Igf1 and Igf2 in FLEF [67] and diminished bioavailability of Igf1 and Igf2 for IGF-signaling [66]. This is in line with the higher presence of Igf1 in FLEF (although not significant), and the detection of Igf2 in only one of the BMEF samples and at higher levels in all four FLEF samples (Table S3). In addition, we found Igf2bp1 to be upregulated in fetal cells upon *MLL::ENL* expression, and unchanged in adult cells (Fig. 4C), likely decreasing the intracellular levels of Igf2 in fetal pre-leukemic cells by repression of Igf2 mRNA translation [68]. Together, our results indicate that adult LICs alter IGF-signaling through upregulation of Igf2r, while fetal LICs have modulated IGF-signaling through extrinsic regulation of ligand availability at steady state, as well as intracellular regulation of Igf2 expression. Treatment of fetal and adult pre-leukemic cells with Igf2 in culture had minor effects on cellular output (Fig. S10A, SB). However, in the absence of oncogene expression, Igf2 appeared to exert a mild anti-differentiation effect on fetal normal cells and a pro-differentiation effect on adult normal cells (Figs. S10A–C). Interestingly, the infant leukemia cell lines KOPN-8 and THP-1 both showed decreased viability in the presence of IGF2, while adult leukemia cells (Mono-Mac-6 and NOMO-1) were unaffected (Figs. 8A, B, and S10E). This effect was distinct from alterations in IGF2R surface expression (Fig. S10F, G), and a short and moderate blocking of the IGF2R prior to addition of IGF2 to the cultures was not sufficient to reverse the observed effect (data not shown). Nevertheless, phospho-proteome analysis confirmed the preferential internalization of IGF2 by IGF2R (Fig. 8C), with no evidence of classical phospho-signaling through IGF1R or insulin receptor (Fig. S11A, B).

Because of this specific viability effect of IGF2 on infant leukemia cells, we further investigated the potential effects to their secretome (Fig. S10D). Intriguingly, both cell lines showed an intensified secretion upon IGF2 treatment (Fig. S11C), and the proteins with induced secretion largely related to neutrophil degranulation, including many cathepsins as well as ELANE, MPO and PRTN3 (Fig. 8D). By mapping our fetal mouse pre-leukemic secretome to the human infant leukemia secretome, we observed the reversal of secretion of these proteins with the addition of IGF2 to the human leukemia cells (Fig. 8E, F). Collectively, the analyses highlight IGF2 bioavailability as a potential point of vulnerability in infant MLLr leukemia.

## Discussion

In this work, we have characterized the functional and proteomic events governing the first stages of leukemic transformation in fetal- and adult-derived LMPPs. In addition, we have described the proteomic composition of the extracellular milieu of the potential LICs and connected that to their protein secretion, as well as to secretion from human leukemic cells. Our work has uncovered ontogenically conserved as well as developmentally regulated molecular signatures of fetal- and adult-derived *MLL::ENL*-mediated leukemia, as well as highlighted an interplay of intra- and extracellular factors in determining disease phenotype and progression.

Our analysis of the in vitro lineage potential of fetal and adult *MLL::ENL*-expressing LMPPs shows an intrinsically programmed lymphoid bias in *in utero*-derived lymphomyeloid progenitor cells, which is in line with our previous findings regarding a diminished capacity for myeloid differentiation in fetal relative to adult LMPPs [11]. In vivo, however, both fetal and adult LMPPs give rise to AML in adult mice. Although translocations of the MLL gene in humans frequently gives rise to lymphoid leukemia [7], most transplantation-based mouse models of MLLr leukemia, including the model utilized in this study, exclusively develop AML in vivo [15, 19, 23, 24]. In line with recent work highlighting transplantation-related stress as a potential cause for this bias [19], donor cells in neonatal recipients, that were conditioned considerably less harshly than adult recipients in our transplantation assay, showed more balanced output. Furthermore, the developmental stage of the niche in which leukemia is propagated has recently been identified as a strong determinant of MLLr leukemia phenotype [20]. Although neonatal recipients failed to develop leukemia during the assayed time course of our transplantation experiment, only fetal *MLL::ENL*-expressing cells were capable of long-term engraftment and multilineage contribution in a neonatal environment, further corroborating that cell-intrinsic and extrinsic factors synergize during MLLr leukemia development and maintenance. In line with this, survival appeared worse in adult recipients of adult cells compared to animals transplanted with fetal cells. This difference could additionally be attributed to fetal-specific RNA-binding protein Lin28b acting as a barrier towards development of *MLL::ENL*-driven AML [69].

Our proteome data points towards differentiation arrest, a hallmark feature of cancer [70], as an ontogenically conserved early event in *MLL::ENL*-mediated transformation. Importantly, we identify several age-specific events occurring during early leukemogenesis, including differences in cell identity following transformation where fetal cells appear to have a more lymphoid-like and immature phenotype compared to adult cells, which instead retain more myeloid features upon induced expression of the oncogene. We additionally show that proteins involved in HDAC signaling, apoptosis, and translation exhibit ontogeny-specific changes in expression upon transformation, indicating potential differences in the vulnerability of the cells to modulation of these processes. Overall, several highlights in our data point to that fetal LMPPs more rapidly acquire leukemic molecular features than adult cells. This may partly explain the cells’ higher susceptibility to MLLr leukemia transformation without secondary mutation. In particular, fetal MLLr cells may be more sensitive to endoplasmic reticulum (ER) stress than their adult counterpart, as translation-related processes were strongly enriched among proteins significantly upregulated in fetal pre-leukemic versus WT cells. In addition, in homeostatic settings, fetal HSPCs have a higher tolerance for translation-associated ER stress than adult cells [42]. Our data suggest that like normal adult HSCs, adult LSCs may require a low translation rate to maintain function, whereas fetal pre-leukemic cells are capable of increasing translation rate beyond its already high baseline level. Our findings regarding elevated expression of Hdac3 and Rbbp4 in fetal but not adult pre-leukemic cells compared to WT may also have implications for the design of treatment schemes involving HDAC inhibitors for infant and adult leukemia.

Considering that normal fetal and adult HSPCs are hallmarked by a high expression and activity of translation-associated and inflammatory proteins, respectively [10, 11], our findings here indicate that oncogenes such as *MLL::ENL* may hijack and amplify these differences to maintain and drive leukemia at the respective ontogenic stages. In addition, some pre-leukemic features that show ontogeny-specific changes upon *MLL::ENL* expression extend to the microenvironment of the LICs. This was particularly prominent regarding metabolic processes, which were suppressed uniquely in the adult *MLL::ENL*-expressing cells, and the BMEF showing lower presence of diverse metabolic proteins compared to FLEF. In addition, we found differential regulation of Igf2 bioavailability and VLA-4 dimer interactions in fetal and adult pre-leukemic cells. The ex vivo assays emphasized that extracellular factors that are present in different quantities in the microenvironments of fetal and adult LICs play a role in the cells’ ability to communicate with their surroundings. Critically, differential dependency on the Igf2/Igf2r and Fn1/VLA-4 axes upon initiation of fetal- and adult-origin MLLr leukemia may represent an opportunity for targeting ontogeny-specific vulnerabilities in *in utero*-derived and adult-onset leukemia.

A striking finding was the alterations to secretion of proteins relating to neutrophil degranulation, and the co-induction of neutrophil and platelet-associated defense response of adult cells upon *MLL::ENL* induction. The release of these proteins from neutrophils or platelets is recognized as first-line defense mechanisms of the immune system [71]. We have previously shown the presence of neutrophil granule proteins already from the short-term HSC stage of adult hematopoiesis [72] and a very low expression in fetal HSPCs [10]. Our findings here, that such first-line defenders of immunity are released from pre-leukemic and leukemic cells, and largely contribute to the cells’ ontogenic features of communication with their surroundings, is intriguing. Moreover, we found that the secretion of neutrophil degranulation proteins is influenced by both intra- and extracellular ontogeny-specific factors. Future studies will be necessary to better understand the impact of their secretion on leukemia progression.

Collectively, our study sheds light on the molecular underpinnings that are responsible for different disease phenotypes in infant and in adult MLLr leukemia. The identified differential proteomic features that we have revealed here represent vulnerabilities of disease biology and important opportunities for exploration of novel age-tailored therapies to improve the outcome of MLLr leukemia patients.

## Methods

Details of the methods are provided in the Supplemental Information.

### Mice

The inducible mouse model of *MLL::ENL*-driven leukemia (*Col1a1*^TetO_MLL-ENL^, CD45.1) has been described previously [23]. Wild-type C57Bl/6 N mice (CD45.2) were purchased from Taconic Biosciences or bred in-house. All experiments involving animals were performed in accordance with ethical permits approved by the Swedish Board of Agriculture.

### Flow cytometry and FACS

Fetal and adult LMPPs, from E14.5 FL and ABM, respectively, were FACS-sorted as previously described [11]. For proteomics, fetal and adult LMPPs co-cultured for 4 days with OP9 cells were collected and surface-stained with fluorophore-conjugated antibodies against cKit, Flt3, CD11b, CD19, Ly6G, B220, NK1.1 and CD11c. 7AAD was added shortly before analysis to exclude dead cells. All flow cytometry and FACS experiments were performed at the FACS Core Facility at Lund Stem Cell Center.

### In vivo leukemia analysis

A volume corresponding to 2000 *MLL::ENL* LMPPs and 300,000 WT support cells was injected intravenously via the facial vein (neonates) or tail vein (adults) into WT recipients. Disease progression was assessed in PB every 3 weeks starting from 4 weeks post-transplantation by Sysmex and flow cytometry.

### Culture assays

For coculture experiments, fetal and adult WT and iMLL::ENL LMPPs were FACS-sorted into 48-well plates onto pre-established layers of 10,000 OP9 cells per well. For suspension culture experiments, fetal and/or adult iMLL::ENL LMPPs were FACS-sorted into 96-well plates. *MLL::ENL* expression was induced by addition of 1 μg/ml doxycycline hyclate (DOX; Merck). Recombinant mouse or human IGF2 (R&D Systems) was added at 50 or 2000 ng/ml (to mouse cells) and 2000 ng/ml (to human cells). Recombinant Fn1 (R&D Systems) was added at 6 μg/ml and Fbln1 (R&D Systems) was added at 2 μg/ml.

### Proteomic sample preparation and mass spectrometry

Cell pellets corresponding to 40,000 cells were processed using in-StageTip (iST) NHS sample preparation kit (PreOmics) in accordance with manufacturer’s protocol. Digested peptides were labeled using TMTpro reagents (Thermo Scientific). Following desalting, labeled peptides were combined and high-pH-reverse phase (HpH-RP) pre-fractionation was carried out as previously described [11, 30].

The EF collection protocol was modified from previous procedures [49, 73]. One femur and one tibia per mouse (WT) were punctured at both ends, and placed on in-house made ‘3-trap’ tubes. 50 µl sterile PBS was added to the inner tube, and the samples were centrifuged at 300 × *g* for 5 min and an additional 2 min at 500 g without bones before collecting the BMEF. FLs from 9 WT embryos (E14.5) were washed with sterile PBS and placed into a 40 µm cell strainer. 50 µl sterile PBS was injected into the FL before centrifugation at 400 × *g* to obtain the FLEF. BMEF and FLEF samples were cleared at 2000 g, snap frozen and kept at −80 °C for further processing. The nine FLEF samples were pooled into four samples. BMEF and FLEF were concentrated with Amicon 3 K filters. Concentrated EF samples were diluted in 50 mM ammonium bicarbonate (Sigma) with 0.1% RapiGest (Waters), proteins digested overnight with trypsin (Promega), and the resulting peptides were desalted.

For the secretome analysis, conditioned media was collected from fetal LMPP PVA cultures on day 6, and from adult counterparts on day 8. Four volumes of acetone were added to the collected media to precipitate the proteins. Samples were diluted in 50 mM ammonium bicarbonate and proteins digested overnight with trypsin.

Phosphopeptide enrichment was performed as described previously [6] using the Pierce High-Select Fe-NTA Phosphopeptide Enrichment Kit (Thermo). The unbound fraction and washes (flow-through) from the enrichment were combined for the corresponding proteome analysis.

LC–MS analyses were carried out on an Orbitrap Exploris 480 MS instrument equipped with FAIMS Pro and coupled to a reverse phase UltiMate 3000 UHPLC system (all Thermo Fisher Scientific). For TMTpro labeled peptides, data acquisition was carried out in data-dependent mode, and EFs, secretome, phospho-enriched, and phospho-unbound (flow-through/proteome) samples were analyzed by data-independent acquisition (DIA).

### MS raw data processing, protein identification, and statistical analysis

The MS raw files from the TMTpro experiment were searched in Proteome Discoverer (version 2.5, Thermo Scientific). The false discovery rate for peptide-spectrum matches (PSMs) was set to 0.01 using the Percolator node. The MS data of the single‐shot EF samples, secretome, proteome, and phospho-proteome of human cell lines were searched with ‘directDIA’ in Spectronaut (version 17 and 18, Biognosys AG). The Q‐value thresholds were set to 0.01 at PSM, peptide, and protein levels.

Statistical analysis of the TMTpro quantification was performed using MSStatsTMT (version 2.4.1) [74] in R. Differential expression analysis was performed using moderated *t*-tests with Benjamini–Hochberg (BH) multiple hypothesis correction. Proteins with adjusted p-value less than 0.05 between *MLL::ENL* and WT in fetal or adult were considered as differentially expressed. For the EF and secretome DIA data, statistical analysis was performed with MSstats (version 4.4.1 and 4.8.7) [75] using a linear mixed-effect statistical model. The phospho-enriched and phospho-unbound cell line data were statistically analyzed with MSStatsPTM (version 2.4.1) [76] using separate linear mixed-effect statistical model. The BH method was used to account for multiple testing. Differentially expressed proteins were selected with adjusted *p*-value less than 0.001 and a fold change of more than 2 between FLEF and BMEF, adjusted p-value < 0.05 for the secretome, and adjusted *p*-value < 0.05 and a fold change of more than 1.5 for the phospho-proteome analysis.

### Statistical analysis

For all other experiments, differences between groups were assessed by two-tailed Students’ *t*-test (two groups) or one-way ANOVA with Tukey’s *post hoc* test (three or more groups) using Prism software version 9 (GraphPad). Error bars represent SD. ****p < 0.0001, ***p < 0.001, **p < 0.01, and *p < 0.05 and ns not significant. The exact sample size for each experimental group/condition is stated in the figure legends. Samples sizes were predetermined heuristically.

### Supplementary information

Supplemental Information

Table S1

Table S2

Table S3

Table S4

Table S5

Table S6

Table S7

Table S8

## Data Availability

The mass spectrometry proteomics data have been deposited to the ProteomeXchange Consortium via the PRIDE partner repository with the dataset identifiers PXD042249, PXD042251, PXD049014, and PXD049016.
