# Supplementary material for: A complex interplay of intra- and extracellular factors regulates the outcome of fetal- and adult-derived MLL-rearranged leukemia
Source: Leukemia. 2024 Mar 30;38(5):1115–30. doi: 10.1038/s41375-024-02235-5 (PMC11073998; doi:10.1038/s41375-024-02235-5)

SUPPLEMENTARY INFORMATION

A Complex Interplay of Intra- and Extracellular Factors Regulates the Outcome of Fetal- and Adult-Derived MLL-Rearranged Leukemia

Maria Jassinskaja*, Sudip Ghosh*, Joanna Watral, Mina Davoudi, Melina Claesson Stern, Ugarit Daher, Mohamed Eldeeb, Qinyu Zhang, David Bryder and Jenny Hansson

*These authors contributed equally.

List of Supplementary Information

Supplementary Methods, Supplementary Table Legends (Tables S1-S8), Supplementary Figure Legends, Supplementary Figures 1–11.

SUPPLEMENTARY METHODS

Experimental Model and Subject Details

Mice

The inducible mouse model of *MLL::ENL*-driven leukemia (*Col1a1*$^{\text{TetO\_MLL-ENL}}$, CD45.1) has been described previously[1]. Wild-type (WT) C57Bl/6N mice (CD45.2) were purchased from Taconic Biosciences or bred in-house. All experiments involving animals were performed in accordance with ethical permits approved by the Swedish Board of Agriculture. Animals were housed in individually ventilated cages (IVC) and provided with sterile food and water *ad libitum*. Adult mice used in all experiments were 6-12 weeks old, except for analyses of CD29, CD49d, and CD79a cell surface expression and Igf2, Fn1 and Fbln1 supplement (34-45 weeks). E14.5 embryos and neonatal mice were obtained by timed pregnancies overnight. The morning after mating was considered E0.5.

Human Cell lines

Human leukemic cell lines, THP-1 (*MLL::AF9*, infant AML), KOPN-8 (*MLL::ENL*, infant ALL), NOMO-1 (*MLL::AF9*, adult AML), and Mono-Mac-6 (*MLL::AF9*, adult AML) were obtained from DSMZ.

Cell culture

For co-culture assays, OP9 murine stromal cell lines were stored at -150 ºC in freezing media containing 10% DMSO (Merck). Cells were thawed by briefly placing vials in a water bath at 37 ºC. Cells were seeded at 300,000-500,000 cells per flask in 80 or 175 cm^3 culture flasks (Thermo Scientific) in 20 mL OptiMem + GlutaMAX media (Gibco) supplemented with 10% fetal calf serum (FCS; HyClone), 1% penicillin/streptomycin (Gibco) and 0.02% 50 mM 2-mercaptoethanol (Gibco; hereafter termed complete medium). Cells were maintained in an incubator at 37 ºC and 5% CO_2 and passaged upon reaching 80% confluency (approximately every 48-72 hours). Passaging of cells was performed by incubation with trypsin (HyClone) for 4 min at 37 ºC. Flasks were washed twice with complete medium to retrieve detached cells prior to centrifugation for 5 min at 400 g at room temperature (RT) and reseeding.

For human cell lines THP-1, KOPN-8, and NOMO-1 cells were seeded at 200,000 cells per well in 96-well plates and cultured in RPMI 1640 (Gibco) supplemented with 10% heat-inactivated FCS (HyClone), 1% penicillin/streptomycin (Gibco) whereas Mono-Mac-6 cells were cultured additionally with 2 mM L-glutamine, 1% non-essential amino acids (Gibco), 1 mM sodium pyruvate (Gibco), and 0.1% human insulin (Sigma Aldrich). Cells were maintained in an incubator at 37 ºC and 5% CO_2 and passaged upon reaching 80% confluency.

Method Details

Flow cytometry and FACS

Fetal and adult LMPPs were FACS-sorted as previously described[2]. Briefly, ABM was extracted from hind limbs, hip bones, forelimbs, shoulders, sternum and spine collected in Hank's Balanced Salt Solution (HBSS; HyClone) supplemented with 0.5% bovine serum albumin (BSA) and 2 mM EDTA (hereafter termed collection media). Single-cell suspension of ABM was obtained by crushing bones using a mortar and pestle and passing cell suspensions through a 40 μm filter. Lineage-positive cells were removed from ABM cell suspension by depletion for Gr-1 (RB6-8C5; BioLegend), Ter119 (TER119; BioLegend), CD3 (145-2C11; BioLegend), B220 (RA3-652; BioLegend) and CD11b (M1/70; BioLegend) using biotin-conjugated antibodies with MACS anti-biotin beads using the autoMACS Pro Separator (Miltenyi Biotech) or LS columns (Miltenyi Biotech). Fetal livers (FLs) were extracted from embryos collected at E14.5 gestation into collection media. Single-cell suspension of FLs was obtained by mechanical dissociation and passing through a 40 μm filter. Red cells were removed from FL cell suspensions by depletion for Ter119 using a biotin-conjugated antibody with MACS anti-biotin beads using the autoMACS Pro Separator or LS columns. FL and ABM cells were surface-stained with fluorophore-conjugated antibodies against either Sca-1 (D7; BioLegend), c-Kit (2B8; BioLegend), Gr-1, Ter119, CD3, B220, Flt3 (A2F10; BioLegend), IL-7Rα (A7R34; BioLegend) and CD150 (TC15-12F12.2; BioLegend). For FACS-sorting of pre-leukemic cells for proteomics, fetal and adult LMPPs co-cultured for 4 days with OP9 were collected and incubated with anti-CD16/32 (FC-block; BD Biosciences) prior to surface-staining with fluorophore-conjugated antibodies against cKit, Flt3, CD11b, CD19 (1D3/CD19; BioLegend), Ly6G (1A8; BioLegend), B220, NK1.1 (PK136; BioLegend) and CD11c (N418; BioLegend). Fluorophore-conjugated antibodies against mouse CD29 (HMβ1-1), CD49d (R1-2), and CD79a (F11-172) for flow cytometric analysis were purchased from BioLegend. Fluorophore-conjugated antibodies against human IGF2R (2G11) were obtained from Novous Biologicals, and human CD49d (FAB1354G) and human CD29 (FAB17781P) from R&D

Systems. In all flow cytometry and FACS experiments, cells were incubated with 7AAD (Merck) briefly before analysis to exclude dead cells. All flow cytometry and FACS experiments were performed on BD FACSAriaIIu (70 µm nozzle), BD FACSAriaIII (70 or 85 µm nozzle), BD LSRFortessa or BD LSRFortessa X-20 instruments at the FACS Core Facility at Lund Stem Cell Center. Data analysis was performed in FlowJo (BD).

Sample preparation for cellular proteome analysis

FACS-sorted cells were collected in ice-cold Hank's Balanced Salt Solution (HBSS), centrifuged, and stored as dry pellets at -80 °C until further use. Pellets corresponding to 40,000 cells were processed using in-StageTip (iST) NHS sample preparation kit (PreOmics) in accordance with manufacturer's protocol. Digested peptides were labelled using TMTpro reagents (Thermo Scientific). Immediately before use, TMTpro reagents were equilibrated to room temperature (RT). Vials containing 0.5 mg of TMT label were dissolved in 20 µl of anhydrous acetonitrile (ACN). Labelling was performed by addition of 5 µl dissolved TMTpro to each sample and incubation for 1 hour at RT. Following desalting, labelled peptides were combined and dried by vacuum centrifugation. High-pH-reverse phase (HpH-RP) pre-fractionation was carried out as previously described[2,3]. Fractionated samples were dried by vacuum centrifugation and stored at -20 °C until further use. Prior to LC-MS analysis, samples were dissolved in MS loading buffer (4% ACN/0.1% formic acid (FA)).

Sample preparation for EF proteome analysis

The EF collection protocol was modified from previous procedures[4,5]. One femur and one tibia per mouse (WT) were punctured at both ends, placed on in-house made '3-trap' tubes of a PCR tube, a 0.5 ml tube, and a 1.5 ml tube, where the bottoms of the PCR tube and the 0.5 ml tube were punctured with a 21G needle. 50 µl sterile PBS was added to the PCR tube, and the

samples were centrifuged at low speed (300 g) for 5 minutes at 4°C. Bones were taken out from the PCR tube and the '3-trap' tubes were centrifuged for another 2 minutes at 500 g, 4°C to collect the BMEF. FLs from 9 WT embryos (E14.5) were washed with sterile PBS and placed into a 40 μm cell strainer. 50 μl sterile PBS was injected into the FL before centrifugation at 400 g for 10 minutes at 4°C to obtain the FLEF. BMEF and FLEF samples were next cleared at 2000 g for 20 minutes at 4°C, snap frozen and kept at -80°C for further processing. Protein concentration was estimated with BCA assay (Thermo Scientific). The nine FLEF samples were pooled into four samples. BMEF and FLEF samples were transferred into Amicon 3K filters and concentrated at 14000 g, room temperature until 20 μl of concentrated sample remained in the column. Filters were then inverted into new collection tubes and centrifuged at 1000 g for 2 minutes at room temperature. For proteomic analysis, approximately 50 μg of concentrated EF samples were diluted in 50 mM ammonium bicarbonate (Sigma) with 0.1% RapiGest (Waters) to denature proteins and shaken on a thermomixer (Eppendorf) at 400 rpm for 15 minutes decreasing the temperature from 80°C to 56°C, at 400 rpm, followed by reduction of disulfide bonds with 0.1M dithiothreitol at 56°C, cysteine alkylation with 0.2 M iodoacetamide at room temperature, and digestion overnight at 37°C with sequencing grade modified trypsin (enzyme:protein ratio 1:50, Promega). Digested peptides were acidified with 10% trifluoroacetic acid (TFA) and RapiGest was precipitated by incubation at 37°C. Peptides were desalted, dried by vacuum centrifugation, and resuspended in MS loading buffer prior to LC-MS analysis. Peptide concentrations were measured at 280 nm (Nanodrop 2000, Thermo Scientific) and 1 μg peptide was injected for LC-MS/MS analysis.

Polyvinyl alcohol (PVA) culture of LMPPs

FACS-sorted 200 fetal or 1000 adult LMPPs per well were cultured in a round-bottom 96-well plate in serum-free media composed of Ham's F12 media (Gibco), 1% Insulin-Transferrin -

Selenium-Ethanolamine (ITSX, Gibco), 10 mM HEPES, 1% Penicillin-Streptomycin-Glutamine (Gibco), 100 ng/mL murine TPO (Peprotech), 10 ng/mL murine SCF (Peprotech), 1 mg/mL PVA (Sigma Aldrich) and 25 ng/mL Flt3l (Stem Cell Technologies) for adult LMPPs or 10 ng/mL Flt3l for fetal LMPPs. *MLL::ENL* expression was induced by addition of 1 µg/mL DOX. Cells without DOX treatment were seeded as control (also called WT). For Fbln1 and Fn1 assays, 2 µg/mL recombinant human Fbln1 and/or 6 µg/mL recombinant Fn1 (R&D Systems) were added to cultures with DOX. An additional 0.5 µg/mL DOX was added at day 4 of cultures. For fetal LMPPs, media was collected on day 6 of culture, and on day 8 for the adult counterpart. Cells were not passaged during the cultures, and conditioned media were collected without disturbing the cells. PVA media without the cells were collected as negative control of the secretome.

Secretome and phospho-proteome analysis of human cell lines

For the secretome and phospho-proteome analysis of human leukemia cells, THP-1 and KOPN-8 cells were washed three times with PBS and then cultured at $2x10^6$ cells per well in serum-free RPMI 1640 (Gibco) media. Recombinant human IGF2 (R&D Systems) was added at 10 µg/mL to the cultures. Conditioned media were collected 17 hours after treatment and the cells were washed with PBS, and collected into new tubes for the phospho-proteome analysis. Media without the cells were collected as negative control of the secretome.

Sample preparation for secretome analysis

The collected media from the Fbln1 and/or Fn1-treated LMPPs, DOX-treated and untreated LMPPs, media without cells, and IGF2-treated and untreated THP-1 and KOPN-8 cells were centrifuged at 350 g for 5 min at 4°C. The supernatant was transferred to LoBind Tubes and stored at -80°C until further analysis. Four volumes of ice-cold acetone were added to the

samples, vortexed, and incubated for 1 hour at -20°C. The samples were centrifuged at 15,000 g for 10 min at RT and the supernatant was discarded. The precipitated pellets were dissolved in 50 mM ammonium bicarbonate (Sigma) to denature proteins followed by reduction of disulfide bonds with 0.1M dithiothreitol at 56°C, cysteine alkylation with 0.2 M iodoacetamide at room temperature, and digestion overnight at 37°C with sequencing grade modified trypsin (enzyme:protein ratio 1:50, Promega). Digested peptides were acidified with 10% trifluoroacetic acid (TFA), desalted, and dried by vacuum centrifugation. The dried peptides were resuspended in MS loading buffer prior to LC-MS analysis. Peptide concentrations were measured at 280 nm (Nanodrop 2000, Thermo Scientific) and the sample was injected twice.

Sample preparation for phospho-proteome analysis

Phosphopeptide enrichment for the IGF2-treated and untreated THP-1 and KOPN-8 cells was performed as described previously[6] using the Pierce High-Select Fe-NTA Phosphopeptide Enrichment Kit (Thermo) according to manufacturer instructions. The unbound fraction and washes (flow-through) from the enrichment were combined for the corresponding proteome analysis.

OP9/OP9Dl1 co-culture assays

For co-culture experiments, fetal and adult WT and iMLL::ENL LMPPs were FACS-sorted into 48-well plates onto pre-established layers of 10,000 OP9 cells per well. Cells were cultured in complete medium supplemented with 10 ng/mL stem cell factor (SCF), 10 ng/mL Flt3-ligand (Flt3l), 10 ng/mL interleukin (IL)-7, and 5 ng/mL IL-3. All cytokines were purchased from Stem Cell Technologies. *MLL::ENL* expression was induced by addition of 1 µg/ml doxycycline hyclate (DOX; Merck). Cells were passaged and provided with fresh media every 2-3 days.

Suspension culture assays

For suspension culture experiments, fetal and/or adult iMLL::ENL LMPPs were FACS-sorted into 96-well plates. Cells were cultured in complete medium supplemented with 10 ng/mL SCF, 10 ng/mL Flt3l, 10 ng/mL IL-7 and 5 ng/mL IL-3 for 3 or 4 days. *MLL::ENL* expression was induced by addition of 1 µg/ml DOX. For IGF2 assays, recombinant mouse or human IGF2 purchased from R&D Systems was added at 50 or 2000 ng/mL (mouse) and 2000 ng/mL (human) to the cultures, and cells analyzed by flow cytometry at day 4 for mouse cells and day 2 for human cells. To partially block the Igf2r, cells were treated with 2000 ng/ml IGF2R (R&D Systems) for 30 minutes prior to the IGF2-treatment. For the Fn1 and Fbln1 assays, the cells were treated with recombinant human Fbln1 (2 µg/ml; R&D Systems) and/or recombinant human Fn1(6 µg/ml; R&D Systems) and analyzed by flow cytometry at day 4 for mouse cells and day 2 for human cells.

Ex vivo lineage-potential analysis

Starting from day 4 of culture, cells were collected from wells every 3 days for flow cytometric analysis. Prior to flow cytometric analysis, cells were incubated with FC-block and surface-stained with fluorophore-conjugated antibodies against cKit, Flt3, CD11b, CD19, Ly6G, B220, NK1.1 and CD11c.

In vivo leukemia analysis

Fetal and adult *MLL::ENL* LMPPs were FACS-sorted and frozen at -80°C together with unfractionated WT BM in fetal bovine serum (FBS) containing 10% dimethyl sulfoxide (DMSO). For neonatal recipients, pregnant WT females were put on a DOX-containing diet at E18.5. WT adult recipients were put on a DOX-containing diet 4 days prior to transplantation.

Neonatal (24-48 h old) and adult recipients were pre-conditioned by irradiation with 350 and 900 cGy, respectively. Frozen cells were thawed and resuspended in sterile PBS and a volume corresponding to 2000 *MLL::ENL* LMPPs and 300,000 WT support cells was injected intravenously via the facial vein (neonates) or tail vein (adults). Disease progression was assessed in peripheral blood (PB) every 3 weeks starting from 4 weeks post transplantation by Sysmex and flow cytometry. For flow cytometry analysis, red blood cells (RBCs) were lysed by incubating PB cells with ammonium chloride solution for 10 min at RT. After removal of RBCs, PB cells were incubated with FC-block and surface-stained with fluorophore-conjugated antibodies against CD45.1 (A20; BioLegend), CD45.2 (104; BioLegend), CD11b, Ly6G, CD115 (AFS98; BioLegend), B220 and CD3 (145-2C11; BioLegend) prior to flow cytometric analysis. Mice were euthanized when they became moribund. In addition to PB, spleen and BM were collected and analyzed by flow cytometry to determine disease type. Following RBC lysis, spleen and BM cells were incubated with FC-block and surface-stained with fluorophore-conjugated antibodies against CD45.1, CD45.2, CD11b, Ly6G, B220, CD19, cKit, Flt3, CD3 and Ter119 (TER119; BioLegend) prior to flow cytometric analysis. In cases where mice died prior to euthanasia could be performed, spleen size and weight were examined to determine if leukemia was the cause of death. These animals were included in survival analyses but not subjected to flow cytometric analysis due to extensive necrosis in the tissues.

Liquid chromatography and mass spectrometry

LC-MS analyses were carried out on an Orbitrap Exploris 480 MS instrument with a reverse phase UltiMate 3000 UHPLC system via an EASY-Spray ion source equipped with FAIMS Pro (all Thermo Fisher Scientific). For TMTpro labeled peptides, each fraction was injected twice and separated using a 120 min linear gradient separation followed by tandem MS. Injected peptides were loaded onto a trap cartridge (Acclaim PepMap C18, 5 µm, 300 µm x 5

mm, Thermo Fisher Scientific), followed by gradient elution of peptides on an EASY-Spray analytical column (2 µm particle size, 75 µm inner diameter x 500 mm length, Thermo Fisher Scientific) using 0.1% (v/v) FA in LC-MS-grade water (solvent A) and 0.1% FA in 80% ACN (solvent B) as the mobile phases. Peptides were loaded with a constant flow of solvent A at 5 µl/min onto the trapping column and eluted via the analytical column at a constant flow of 300 nl/min. Equilibration of the column was performed for 14 minutes at a 2% concentration of solvent B. During the elution step, the percentage of solvent B was increased in a linear fashion from 2% to 4% in 3 minutes, to 16% in 83 minutes, to 25% in 45 minutes, and finally to 85% in an additional 5 minutes. FAIMS compensation voltage (CV) was set to -40, -60, and -80. The spray voltage was set at 2.1 kV and the ion transfer tube temperature was set at 275°C. Data acquisition was carried out using a data-dependent 'Top speed' MS2-method (1 s/CV). The RF lens was set to 40%. The full MS scan was performed in the Orbitrap in the range of 400 to 1400 m/z at a resolution of 120,000 at full-width-half-max (FWHM) using an automatic gain control (AGC) of 300% and a maximum ion accumulation time of 50 ms. The intensity threshold was set to 5.0 e4 and mass tolerance to 10 ppm. The most intense ions selected in the first MS scan were isolated for higher-energy collision-induced dissociation (HCD) at a precursor isolation window width of 0.7 m/z, an AGC of 200%, a maximum ion accumulation time of 120 ms and a resolution of 45,000 FWHM. The first mass and the normalized collision energy were set to 110 m/z and 32%, respectively.

EFs, secretome, phospho-enriched, and phospho-unbound (flow-through/proteome) samples were analyzed by data-independent acquisition (DIA). Digested peptides were loaded onto a trap cartridge (Acclaim PepMap C18, 5 mm particle size, 0.3 mm inner diameter x 5 mm length, Thermo Fisher Scientific) and separated by EASY-Spray analytical column (2 mm particle size, 75 mm inner diameter x 500 mm length, Thermo Fisher Scientific). Each sample was injected twice and eluted with a linear gradient ranging from 2-25% Solvent B (0.1% FA

in 80% ACN) over 100 min, 25-40% B over 20 min, 40-90% B over 2 min and held at 90% B for 5 min at a constant flow rate of 300 nl/min at 45°C. FAIMS compensation voltages (CV) were set to -45 and -60. The spray voltage was set at 2.1 kV and the ion transfer tube temperature was set at 275°C. For DIA analysis, peptides were analyzed with one full scan (340–1,300 m/z, R = 120,000) at a normalized AGC target of 300%, followed by 29 (EF and phospho-enriched samples) or 20 (secretome and phospho-unbound (proteome) samples) DIA MS/MS scans (350–1,050 m/z) in HCD mode (isolation window 23.4 (EF and phospho-enriched samples) or 35 m/z (secretome and phospho-unbound (proteome) samples), 1 m/z window overlap, normalized collision energy 30%), with fragments detected in the Orbitrap (R = 15,000). All data were acquired in positive polarity and MS/MS were acquired in centroid mode (except for the phospho-enriched samples which were acquired in profile mode).

MS raw data processing and protein identification

The MS raw files from the TMTpro experiment were searched in Proteome Discoverer (version 2.5, Thermo Scientific) against the Swissprot mouse database together with isoforms of the human MLL protein, peptide sequences for the MLL::ENL fusion protein, as well as commonly observed contaminants and reversed sequences for all entries using the Sequest HT node. The enzyme was set to trypsin with up to two missed cleavages. Cysteine acetylhypusinylation, N-terminal and lysine TMTpro were set as static modifications whereas methionine oxidation, N-terminal acetylation, were set as dynamic modifications. TMT batch-specific isotopic correction factor was applied in the reporter ion quantification. MS1 mass tolerance was set to 10 ppm and MS2 to 0.02 Da. The false discovery rate for peptide-spectrum matches (PSMs) was set to 0.01 using the Percolator node. The co-isolation threshold was set to 50. Proteins were quantified based on the average corrected TMT reporter ion intensities from two technical replicates per sample.

The MS data of the single-shot BMEF and FLEF samples, secretome samples, and phospho-proteome samples were searched with 'directDIA' in Spectronaut (version 17 and 18, Biognosys AG) against the mouse or human SwissProt reference proteome along with commonly used contaminants. Searches used carbamidomethylation as fixed modification and acetylation of the protein N-terminus and oxidation of methionines or 'STY' for the phospho-enriched samples, as variable modifications. The Trypsin/P proteolytic cleavage rule was used, permitting a maximum of 2 missed cleavages and a minimum peptide length of 7 amino acids. Data filtering was set to Q-value. 'Cross run normalization' was enabled with Normalization Strategy set to 'local normalization' based on rows with 'Identified in All Runs (Complete)'. Normalization filter type was set to 'Phospho (STY)' additionally for the phospho-proteome analysis. The Q-value thresholds were set to 0.01 at PSM, peptide, and protein levels.

MS statistical analyses and bioinformatics analysis

Statistical analysis of the TMTpro quantification was performed using MSStatsTMT (version 2.4.1)[7] in R. PSM results from Proteome Discoverer were exported and converted into MSstatsTMT-compatible format using 'PDtoMSstatsTMTFormat' function. PSMs were filtered with peptide percolator q-value < 0.01. Only unique peptides were used for protein quantifications. Protein summarization was performed using the 'msstats' method and global median normalization was performed. Differential expression analysis was performed using moderated t-tests with Benjamini–Hochberg (BH) multiple hypothesis correction. Proteins with adjusted p-value less than 0.05 between *MLL::ENL* and WT in fetal or adult were considered as differentially expressed. Principal component analysis (PCA) was performed using the top 300 most variably expressed proteins using the DESeq, statmod and PCAtools packages in R. Mapping of PC loadings to transcriptome data from BloodSpot was carried out

using the ‘‘normal mouse hematopoiesis’’ dataset[34]. A radar plot was generated using min-max scaled median values of marker genes in each cell type.

For the DIA datasets, ‘features’ were exported from Spectronaut for statistical analysis with MSstats[7] (version 4.4.1 and 4.8.7). For the phospho-proteome analysis, phospho-enriched and unenriched datasets were analysed together with MSstatsPTM (2.4.1)[8]. Contaminants were filtered and features were converted to MSStats format for downstream processing. For the secretome datasets, proteins identified in the respective control media were also manually excluded from downstream processing. Uninformative features and outliers were removed (one of six FL WT and one of six FL MLLr) and missing values were imputed for both the proteome and phospho-proteome analysis. Phospho-site level quantification was performed for the phospho-proteome datasets. For the EF dataset, MSStats group comparison was done for FLEF versus BMEF, while for the secretome datasets of fetal and adult LMPPs, the comparisons were *MLL::ENL* versus WT, as well as *MLL::ENL* versus Fn1, *MLL::ENL* versus Fbln1, and *MLL::ENL* versus Fn1+Fbln1. The secretomes of IGF2 treated THP-1 and KOPN-8 cells were compared with the experimental control. The BH method was used to account for multiple testing. Differentially expressed proteins were selected with adjusted p-value less than 0.001 and a fold change of more than 2 between FLEF and BMEF, adjusted p-value < 0.05 for the secretome, adjusted p-value < 0.05 and a fold change of more than 1.5 for the phospho-proteome analysis. Protein or phospho-site level abundance per sample or condition was used for further analysis and plotting. Protein subcellular localization was predicted with DeepLoc 2.0[9] and proteins with annotation ‘extracellular’ were extracted. Gene set enrichment and overrepresentation analysis were performed with ClusterProfiler[10]. Ligand-receptor interactions were curated by mapping the identified intracellular (TMTpro *MLL::ENL* vs WT) and extracellular (BMEF vs FLEF) proteins to the mouse ligand-receptor pairs downloaded from CelltalkDB[11] (http://tcm.zju.edu.cn/celltalkdb/) and Cellinker[12] (http://www.rna-

society.org/cellinker/) database. Receptors were also mapped in our previous study[13] and the corresponding ligands were retrieved. The resulting networks and subnetworks were visualized in Cytoscape[14]. For the secretome data, protein-protein interactions were retrieved from STRING[15].

Statistical analysis

For all other experiments, differences between groups were assessed by two-tailed Students' t-test (two groups) or one-way ANOVA with Tukey's *post hoc* test (three or more groups) using Prism software version 9 (GraphPad). Error bars represent SD. ****$p < 0.0001$, ***$p < 0.001$, **$p < 0.01$, and *$p < 0.05$ and ns = non-significant. No randomization was used, and no blinding was done.

Data availability

The mass spectrometry proteomics data have been deposited to the ProteomeXchange Consortium via the PRIDE partner repository with the dataset identifier PXD042249 (accessed using username reviewer_pxd042249@ebi.ac.uk and password H7WFzbPJ), PXD042251 (accessed using username reviewer_pxd042251@ebi.ac.uk and password Fhr8aH6r), PXD049014 (accessed using username reviewer_pxd049014@ebi.ac.uk and password pO60K2fs), and PXD049016 (accessed using username reviewer_pxd049016@ebi.ac.uk and password 5sxPLybC).

SUPPLEMENTARY REFERENCES

1. Ugale, A. *et al.* Hematopoietic Stem Cells Are Intrinsically Protected against MLL-ENL-Mediated Transformation. *Cell Reports* **9**, 1246–1255 (2014).

2. Jassinskaja, M. *et al.* Ontogenic shifts in cellular fate are linked to proteotype changes in lineage-biased hematopoietic progenitor cells. *Cell Reports* **34**, (2021).
3. Dimayacyac-Esleta, B. R. T. *et al.* Rapid High-pH Reverse Phase StageTip for Sensitive Small-Scale Membrane Proteomic Profiling. *Anal. Chem.* **87**, 12016–12023 (2015).
4. Sun, W. *et al.* Characterization of the liver tissue interstitial fluid (TIF) proteome indicates potential for application in liver disease biomarker discovery. *J Proteome Res* **9**, 1020–1031 (2010).
5. Mittenbühler, M. J. *et al.* Isolation of extracellular fluids reveals novel secreted bioactive proteins from muscle and fat tissues. *Cell Metab* **35**, 535-549.e7 (2023).
6. Schweppe, D. K., Rusin, S. F., Gygi, S. P. & Paulo, J. A. Optimized Workflow for Multiplexed Phosphorylation Analysis of TMT-Labeled Peptides Using High-Field Asymmetric Waveform Ion Mobility Spectrometry. *J Proteome Res* **19**, 554–560 (2020).
7. Huang, T. *et al.* MSstatsTMT: Statistical Detection of Differentially Abundant Proteins in Experiments with Isobaric Labeling and Multiple Mixtures. *Mol Cell Proteomics* **19**, 1706–1723 (2020).
8. Kohler, D. *et al.* MSstatsPTM: Statistical Relative Quantification of Posttranslational Modifications in Bottom-Up Mass Spectrometry-Based Proteomics. *Molecular & Cellular Proteomics* **22**, (2023).
9. Thumuluri, V., Almagro Armenteros, J. J., Johansen, A. R., Nielsen, H. & Winther, O. DeepLoc 2.0: multi-label subcellular localization prediction using protein language models. *Nucleic Acids Research* **50**, W228–W234 (2022).
10. Wu, T. *et al.* clusterProfiler 4.0: A universal enrichment tool for interpreting omics data. *Innovation* **2**, (2021).
11. Shao, X. *et al.* CellTalkDB: a manually curated database of ligand-receptor interactions in humans and mice. *Brief Bioinform* **22**, bbaa269 (2021).

12. Zhang, Y. *et al.* Cellinker: a platform of ligand-receptor interactions for intercellular communication analysis. *Bioinformatics* btab036 (2021) doi:10.1093/bioinformatics/btab036.
13. Jassinskaja, M. *et al.* Comprehensive Proteomic Characterization of Ontogenic Changes in Hematopoietic Stem and Progenitor Cells. *Cell Reports* **21**, 3285–3297 (2017).
14. Shannon, P. *et al.* Cytoscape: A Software Environment for Integrated Models of Biomolecular Interaction Networks. *Genome Res.* **13**, 2498–2504 (2003).
15. Szklarczyk, D. *et al.* The STRING database in 2023: protein–protein association networks and functional enrichment analyses for any sequenced genome of interest. *Nucleic Acids Research* **51**, D638–D646 (2023).

SUPPLEMENTARY TABLE LEGENDS

Table S1. Differential protein expression of proteins in fetal and adult WT and leukemic cells. Related to Figure 3.

Table S2. GSEA enrichment of fetal and adult leukemic vs WT cells. Related to Figure 4.

Table S3. Differential protein expression in extracellular fluids of fetal liver and adult bone marrow. Related to Figure 5.

Table S4. GSEA enrichment of FLEF vs BMEF. Related to Figure 5.

Table S5. Ligand-receptor pairs of cell surface receptors of the cellular leukemia initiation proteomic data and their corresponding ligands in the EF proteome (6A), as well as ligand-receptor pairs of cell surface receptors of the cellular proteomic comparisons of fetal and adult HSPCs and their corresponding ligands in the EF proteome (S6A). Related to Figures 6 and S6.

Table S6. Differential protein expression in the secretome of fetal and adult *MLL::ENL* and WT LMPPs and differential protein expression in the secretome of fetal and adult LMPPs treated with Fbln1 and/or Fn1. Related to Figures 7 and S9.

Table S7. Differential protein expression in the secretome of IGF2 treated and untreated THP-1 and KOPN-8 leukemic cell lines. Related to Figures 8 and S11.

Table S8. Differential phospho-site and protein expression in IGF2 treated and untreated THP-1 and KOPN-8 leukemic cell lines. Related to Figure 8.

SUPPLEMENTARY FIGURE LEGENDS

Figure S1. Temporal cell output of fetal and adult iMLL::ENL LMPPs in culture. (A, B) Frequency of $CD11b^+$ (A) and $CD19^+$ (B) cells derived from fetal and adult *MLL::ENL* induced LMPPs. (C, D) Frequency of $CD11b^+$ (C) and $CD19^+$ (D) cells derived from fetal and adult normal, uninduced LMPPs. Error bars represent SD. N = 4 for all displayed graphs. Related to Figure 1.

Figure S2. Transplantation of fetal and adult iMLL::ENL LMPPs into neonatal and adult recipient mice. (A) FACS profiles from fresh FL and BM (top panels), and frozen and thawed fetal and adult LMPPs (bottom panels). (B) Donor chimerism in peripheral blood of neonatal recipients transplanted with fetal or adult iMLL::ENL LMPPs. (C) Myeloid, B- and T cell chimerism in neonatal recipients transplanted with fetal or adult iMLL::ENL LMPPs. (D, E) Chimerism (D) and cellular composition (E) in the BM of recipients transplanted as neonates. These animals did not succumb to disease. (F) Blood counts for adult recipients of fetal or adult iMLL::ENL LMPPs. (G, H) Chimerism (G) and cellular composition (H) in the BM of adult recipients that did not succumb to disease. FL = fetal liver and ABM = adult bone marrow. Related to Figure 2.

Figure S3. Proteome analysis of fetal and adult WT and iMLL::ENL LMPPs. (A) Pearson correlation between protein expression in different replicates of fetal and adult WT and iMLL::ENL LMPPs. (B) Panther classification of all identified proteins in fetal and adult WT and iMLL::ENL LMPPs. Related to Figure 3 and Table S1.

Figure S4. Correlation in protein expression differences between WT and leukemic cells in fetus and adult. (A) Scatterplot showing average relative expression difference between normal and *MLL::ENL* pre-leukemic cells in fetus and adult. Proteins statistically differentially expressed (adjusted p-value < 0.05) between MLLr and WT in fetus only, adult only, or both, are shown in purple and red, green and blue, and black, respectively. (B) Relative expression difference between normal and *MLL::ENL* cells of proteins whose gene products have previously been identified as upregulated or downregulated in adult pGM cells upon expression of *MLL::ENL*[1] (pGM UP and pGM DN (down), respectively). (C, D) Average log2 fold change of expression of proteins differentially expressed (adjusted p-value < 0.05) between fetus and adult in WT and *MLL::ENL* cells (C) and uniquely differential in WT cells (D). (E) Frequency of $CD79a^+$ cells in cultures of iMLL::ENL fetal and adult LMPPs in the presence of DOX for 4 days. (F) CD79a expression on adult cells following induction of *MLL::ENL ex vivo*. Error bars represent SD. $**p < 0.01$, and $*p < 0.05$. Related to Figure 4 and Table S2.

Figure S5. Details of FLEF and BMEF proteomic analysis. (A) Workflow for the proteomic analysis of fetal liver (FL) and bone marrow (BM) extracellular fluid (EF). Proteins of FLEF and BMEF were extracted as shown in Figure 5A, digested, and analyzed with FAIMS-DIA on Orbitrap Exploris 480. Raw data was processed in Spectronaut in 'library-free' directDIA and statistical analysis was performed in MSStats. (B) Pearson correlation between protein

expression in different replicates of BMEF (left) and FLEF (right). (C) Pearson correlation between average protein expression in BMEF and FLEF. (D) Pipeline for filtering significantly changed proteins between FLEF and BMEF and annotated as 'extracellular'. (E) Heatmap showing the top 10 higher or lower expressed proteins (adjusted p-value < 0.001, fold change > 2) in FLEF and BMEF annotated as 'extracellular'. LFQ = label-free quantification. (F) Hierarchical clustering of the identified proteins in BMEF and FLEF showing the cluster number. Cluster-wise enrichment of biological processes (GO BP) is shown on the right. GeneRatio represent (count of enriched proteins)/(GO BP count). LFQ = label-free quantification. Related to Figure 5 and Tables S3 and S4.

Figure S6. Integrative analysis of the extra- and intracellular proteome of fetal and adult HSPCs. (A) Protein network of extracellular ligands (triangles) of the proteomic comparison of FLEF and BMEF, and cell surface receptors (circles) of the cellular proteomic comparisons of fetal and adult HSPCs[10]. Edges represent known and predicted receptor-ligand-interactions[11,12]. sign. = significant, n.s. = not significant. (B) Protein network of extracellular ligands (triangles) of the proteomic comparison of FLEF and BMEF, and the receptors Itga4 and Itgb1 (circles) of the cellular proteomic comparisons of fetal and adult HSPCs[13]. Edges represent known and predicted receptor-ligand interactions[11,12]. sign. = significant, n.s. = not significant. Related to Figure 6 and Table S5.

Figure S7. Fn1 and Fbln1 treatment of fetal and adult pre-leukemic cells. (A) Workflow for the flow cytometric analysis of Fn1 and/or Fbln1 treatment of fetal and adult *MLL::ENL* expressing cells. (B) Cell surface expression of CD29 and CD49d following treatment of *MLL::ENL* expressing fetal and adult cells with Fn1 and/or Fbln1. (C, D) Proportion of cells expressing CD49d and/or CD29 following treatment of fetal (C) and adult (D) *MLL::ENL*-

expressing cells with Fn1 and/or Fbln1. (E, F) Cellular composition of cultures of adult (E) and fetal (F) *MLL::ENL*-expressing cells treated with Fn1 and/or Fbln1. Error bars represent SD. **p < 0.01, and *p < 0.05. Related to Figure 6.

Figure S8. Fn1 and Fbln1 treatment of human MLLr leukemia cell lines. (A) Workflow for the flow cytometric analysis of Fn1 and/or Fbln1 treatment of human leukemic cell lines THP-1, KOPN-8, Mono-Mac-6, NOMO-1. (B) Viability of THP-1, Mono-Mac-6, and NOMO-1 cells following treatment with Fn1 and/or Fbln1. (C-F) Cell surface expression of CD29 and CD49d on KOPN-8 (C), THP-1 (D), Mono-Mac-6 (E), and NOMO-1 (F) cells following treatment with Fn1 and/or Fbln1. (G-J) Proportion of KOPN-8 (G), THP-1 (H), Mono-Mac-6 (I), and NOMO-1 (J) cells expressing CD29 and/or CD49d following treatment with Fn1 and/or Fbln1. Error bars represent SD. *p < 0.05. Related to Figure 6.

Figure S9. Proteomic analysis of the secretome of fetal and adult pre-leukemic cells upon Fn1 and Fbln1 treatment. (A) Workflow for the secretome analysis of fetal and adult LMPPs with and without *MLL::ENL* induction, and Fn1 and/or Fbln1 treatment of fetal and adult *MLL::ENL* expressing cells. The cells were cultured using albumin-free polyvinyl alcohol (PVA) culture system. Proteins extracted from the secretome samples were digested and analyzed with FAIMS-DIA on Orbitrap Exploris 480. (B) The frequency and count of fetal and adult cells cultured in the PVA media for 6 days (fetal) or 8 days (adult). (C) Heatmap showing the average log2 fold change of all the 'extracellular' annotated proteins between the secretome of MLLr and WT LMPPs of fetal and adult origin. (D) PCA of all extracellular proteins in the secretome of fetal and adult MLLr cells treated with/without Fn1, Fbln1, and Fn1+Fbln1. (E) Heatmap showing the average log2 fold change of 'extracellular' proteins involved in the 'neutrophil degranulation' pathway between the secretome of MLLr and WT cells, and

between untreated MLLr cells and MLLr cells treated with Fn1, Fbln1, or Fbln1+Fn1 in fetal and adult. Significantly changed proteins (adjusted p-value < 0.05) are marked with star. Error bars represent SD. *p < 0.05. Related to Figure 7 and Table S6.

Figure S10. IGF2 treatment differentially affects fetal and adult cells. (A, B) Frequency of immature and mature cell fractions derived from cultures of *MLL::ENL*-induced and uninduced fetal (A) and adult (B) cells with and without addition of Igf2. (C) Proportion of Igf2r$^+$ cells and Igf2r expression in cultures of fetal *MLL::ENL*-induced and uninduced cells with and without addition of Igf2. (D) Workflow for the secretome, proteome and phosphoproteome analysis of THP-1 and KOPN-8 cell lines following IGF2 treatment for 17 hours. Proteins extracted from the secretome samples and enriched for the phosphorylated peptides by Fe-NTA, were digested and analyzed with FAIMS-DIA on Orbitrap Exploris 480. The unbound fraction from the phospho-enrichment was used for global proteome analysis. Cellular readouts of THP-1, KOPN-8, Mono-Mac-6, and NOMO-1 cells treated with IGF2, with or without prior mild blocking of the receptor of IGF2, were performed with FACS analysis at day 2 after treatment. (E) Viability of Mono-Mac-6 and NOMO-1 cells following treatment with IGF2. (F) Proportion of IGF2R$^+$ THP-1, KOPN-8, Mono-Mac-6, and NOMO-1 cells following treatment with IGF2. (G) Cell surface IGF2R expression on THP-1, KOPN-8, Mono-Mac-6, and NOMO-1 cells following treatment with IGF2. Error bars represent SD. ***p < 0.001, **p < 0.01, and *p < 0.05. Related to Figure 8 and Tables S7 and S8.

Figure S11. Phosphoproteome and secretome analysis of IGF2-treated human leukemic cell lines THP-1 and KOPN-8. (A, B) Volcano plots of statistical analysis of phosphorylation changes of IGF2-treated vs untreated THP-1 (A) and KOPN-8 (B) cells. Top 5 up- and down-regulated phosphosites (p-sites) are marked. Phosphosites belonging to proteins involved in

IGF1R pathway, or PI3K-AKT-MTOR signaling are shown in cyan and green. N = 4. (C) Heatmap showing average log2 fold change of all the ‘extracellular’ annotated proteins in the secretome of THP-1 and KOPN-8 with/without IGF2.

SUPPLEMENTARY FIGURES

Supplemental Figure 1

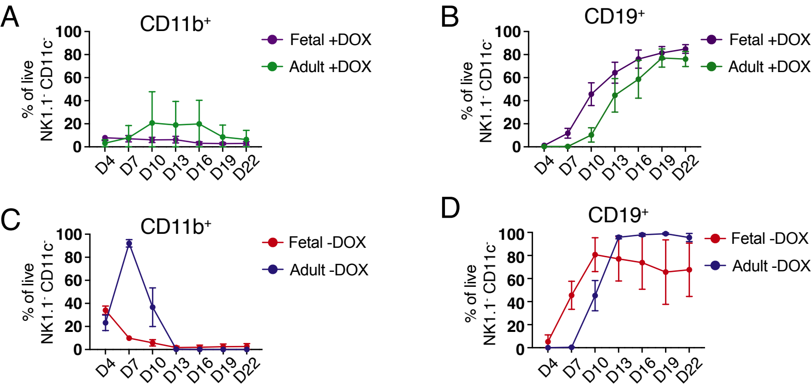

Supplemental Figure 2

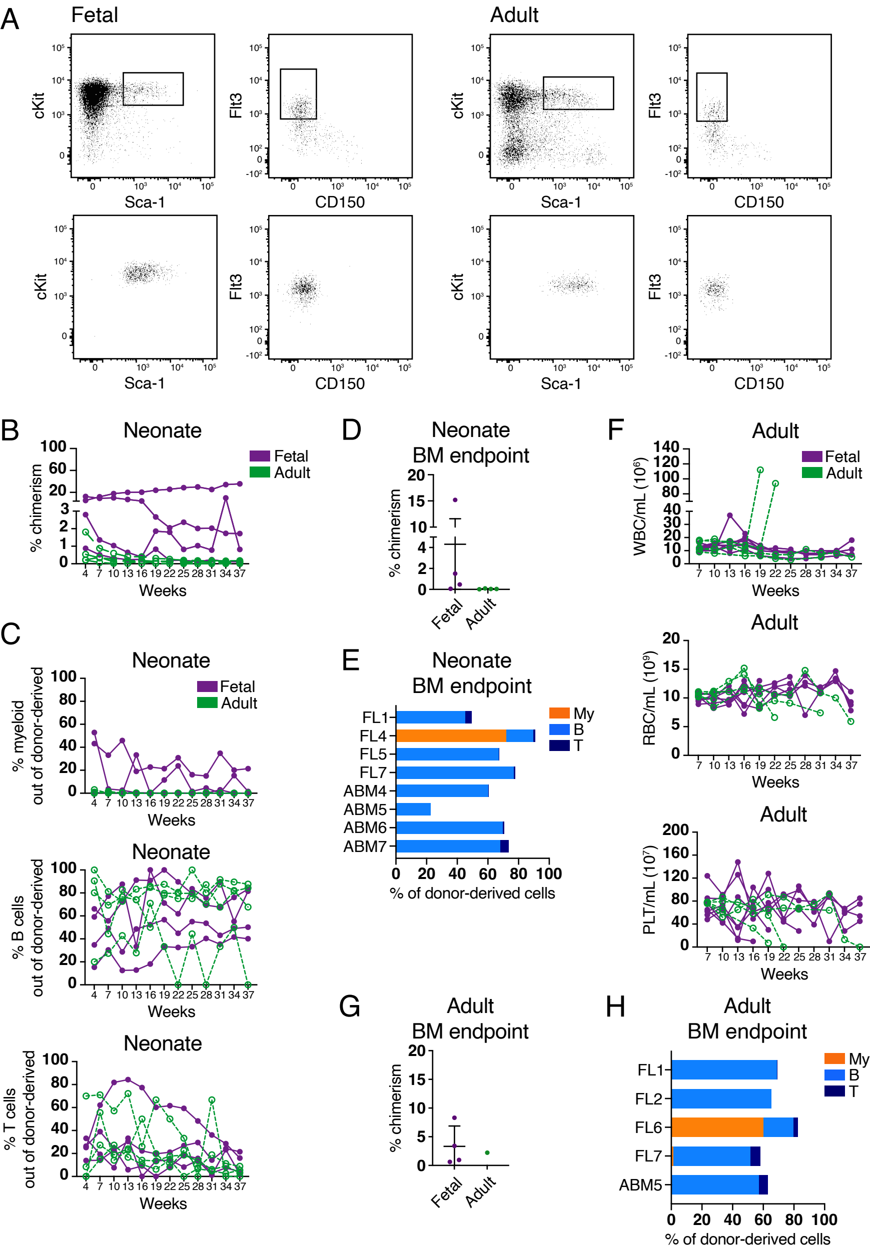

Supplemental Figure 3

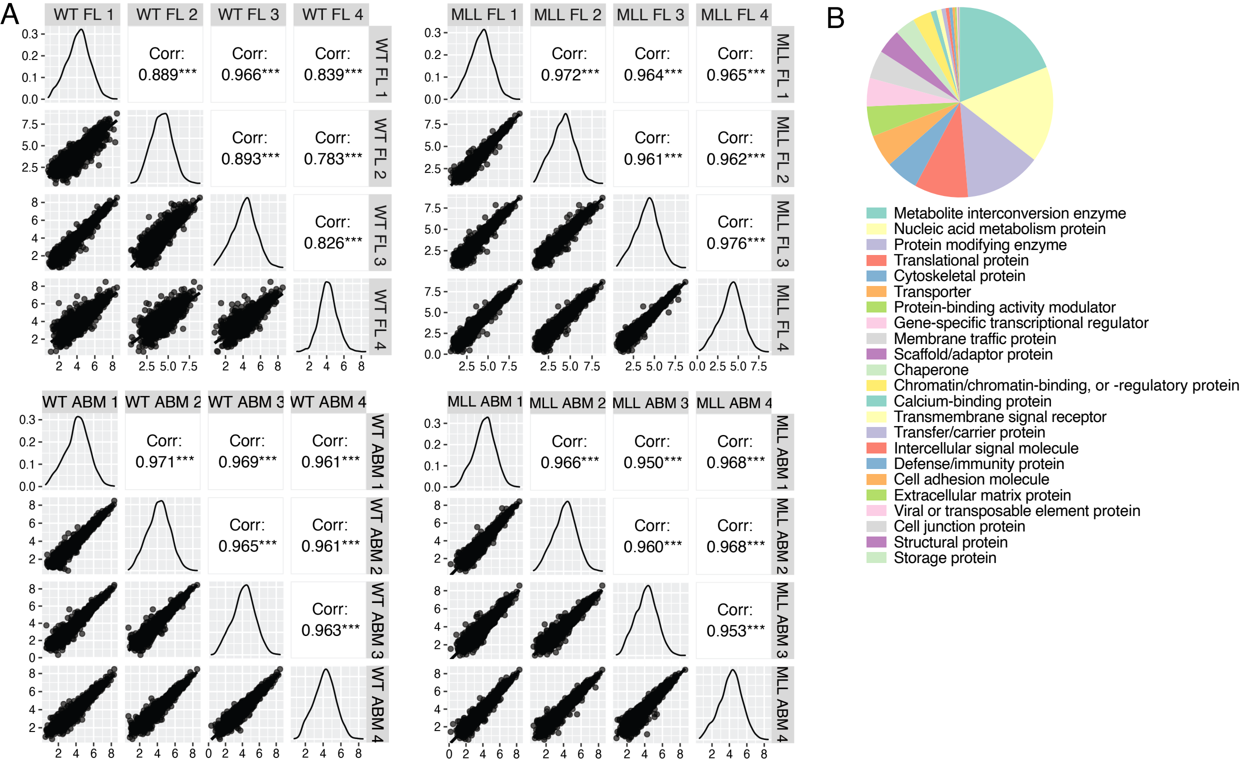

Supplemental Figure 4

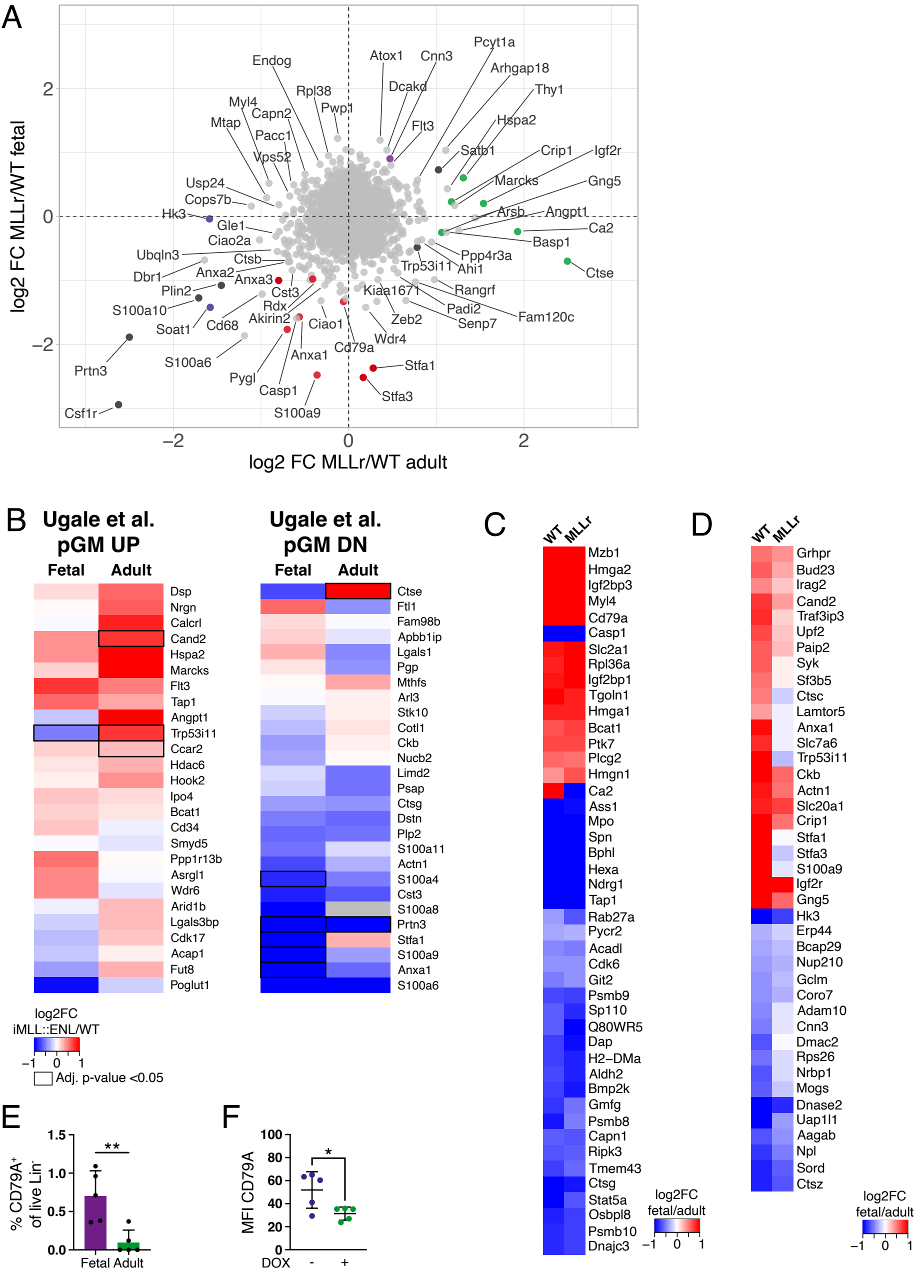

Supplemental Figure 5

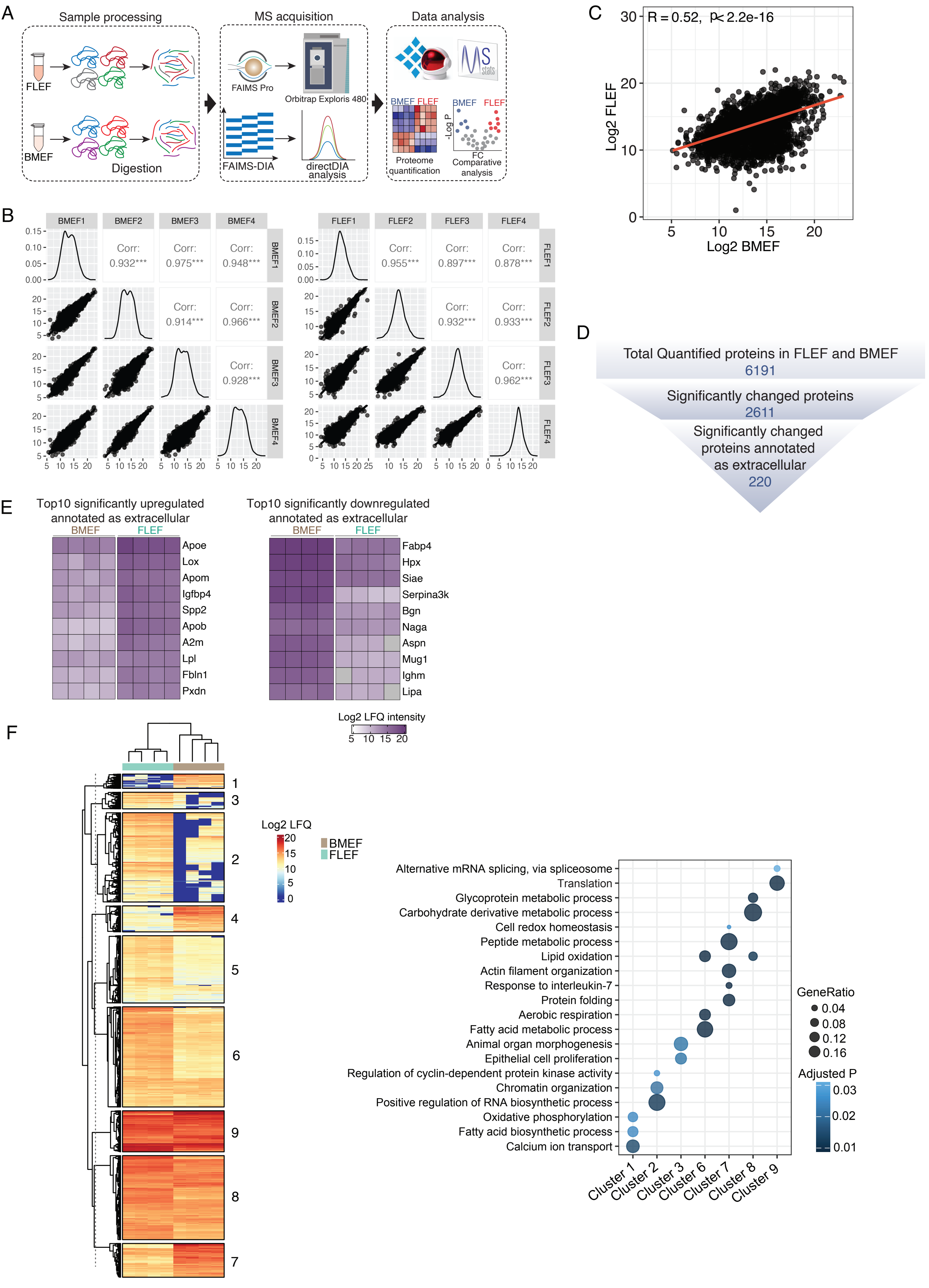

Supplemental Figure 6

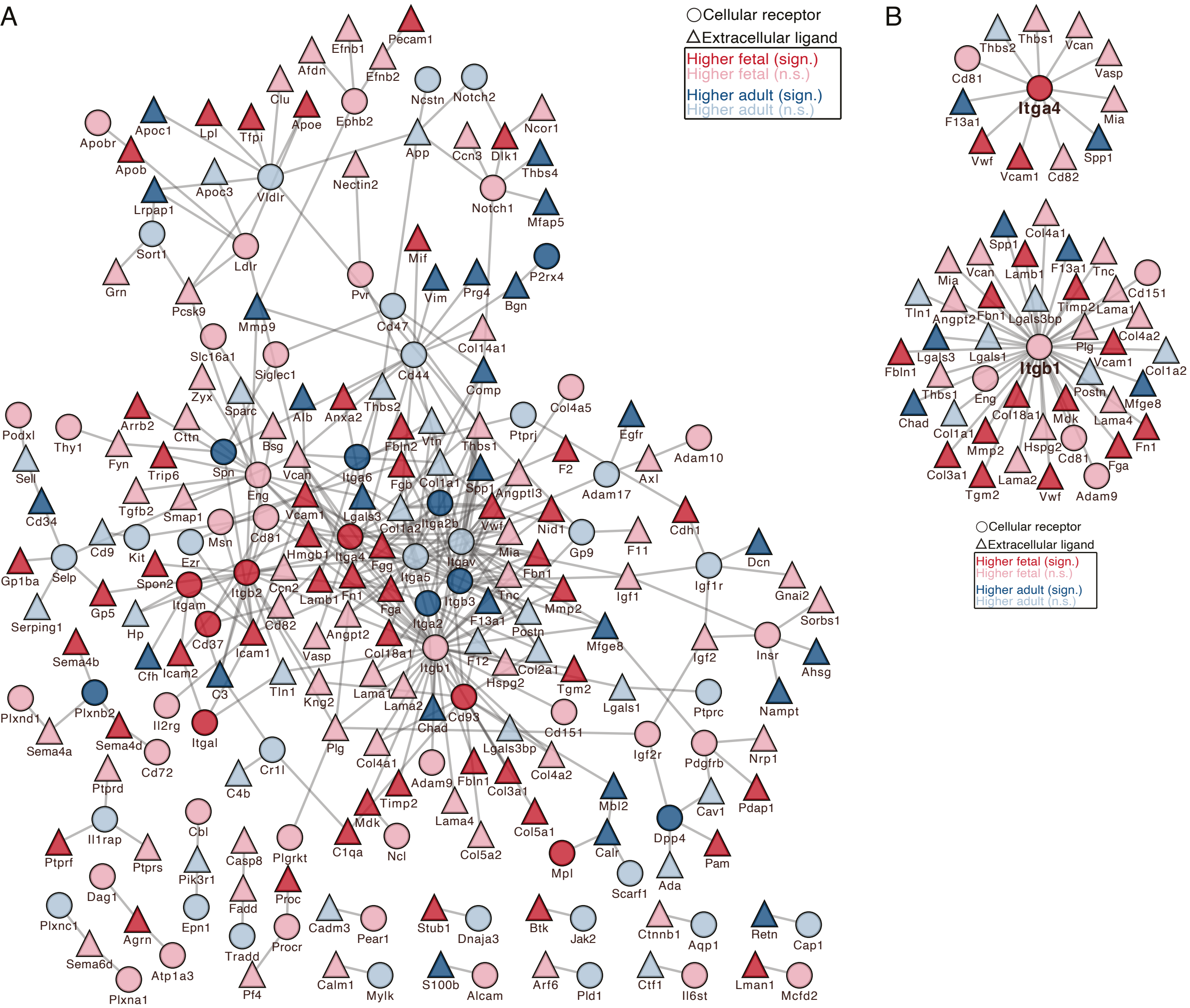

Supplemental Figure 7

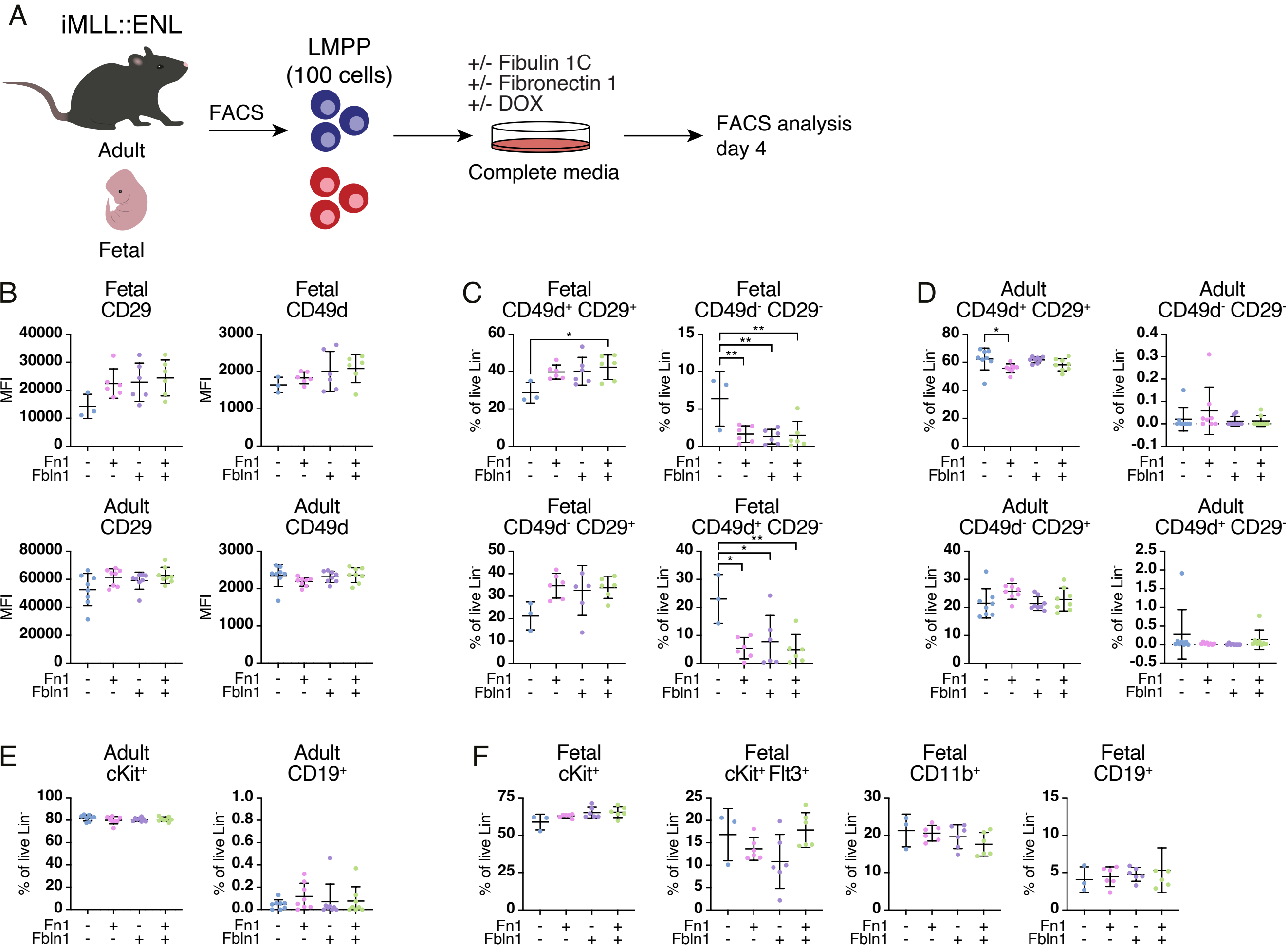

Supplemental Figure 8

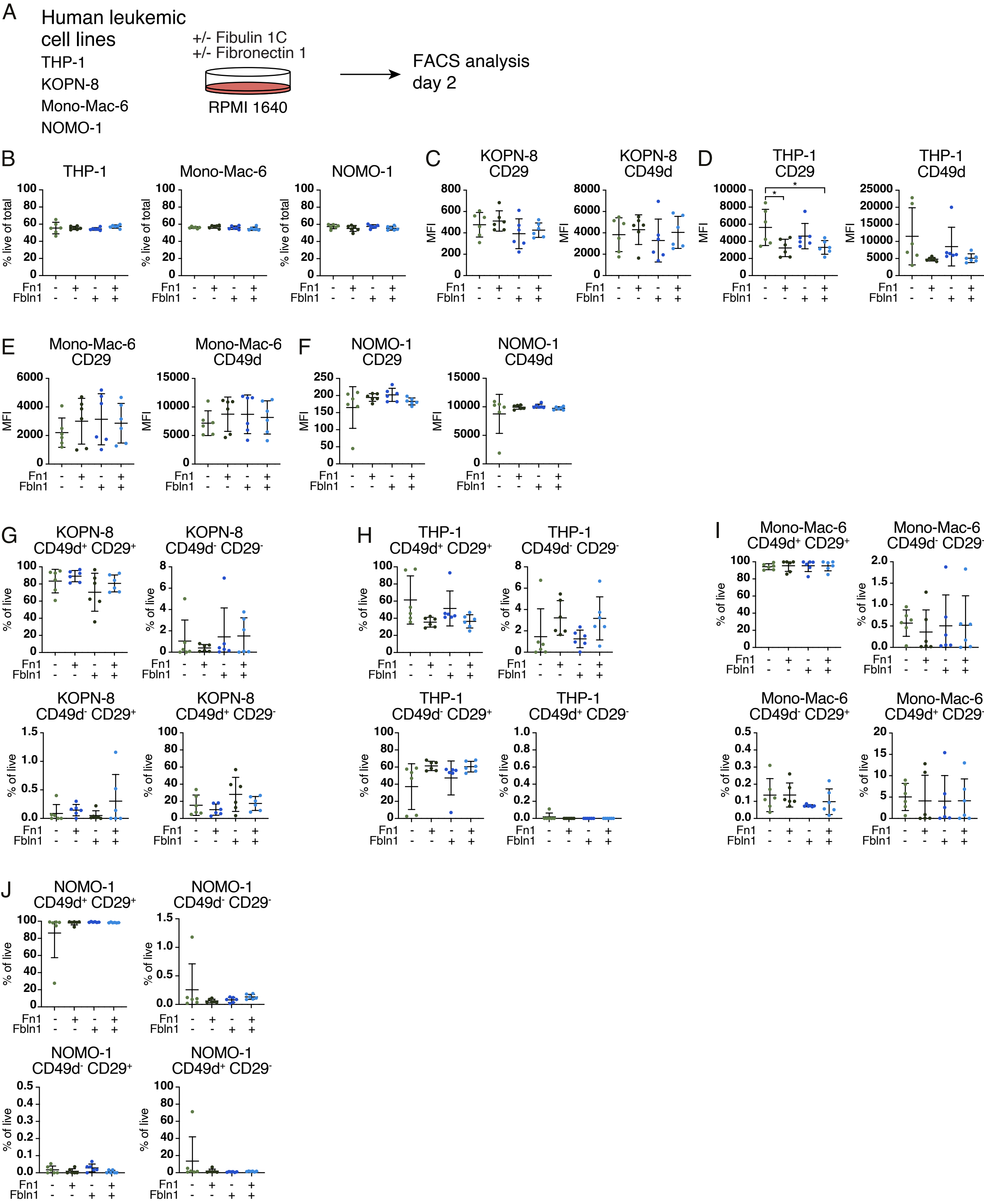

Supplemental Figure 9

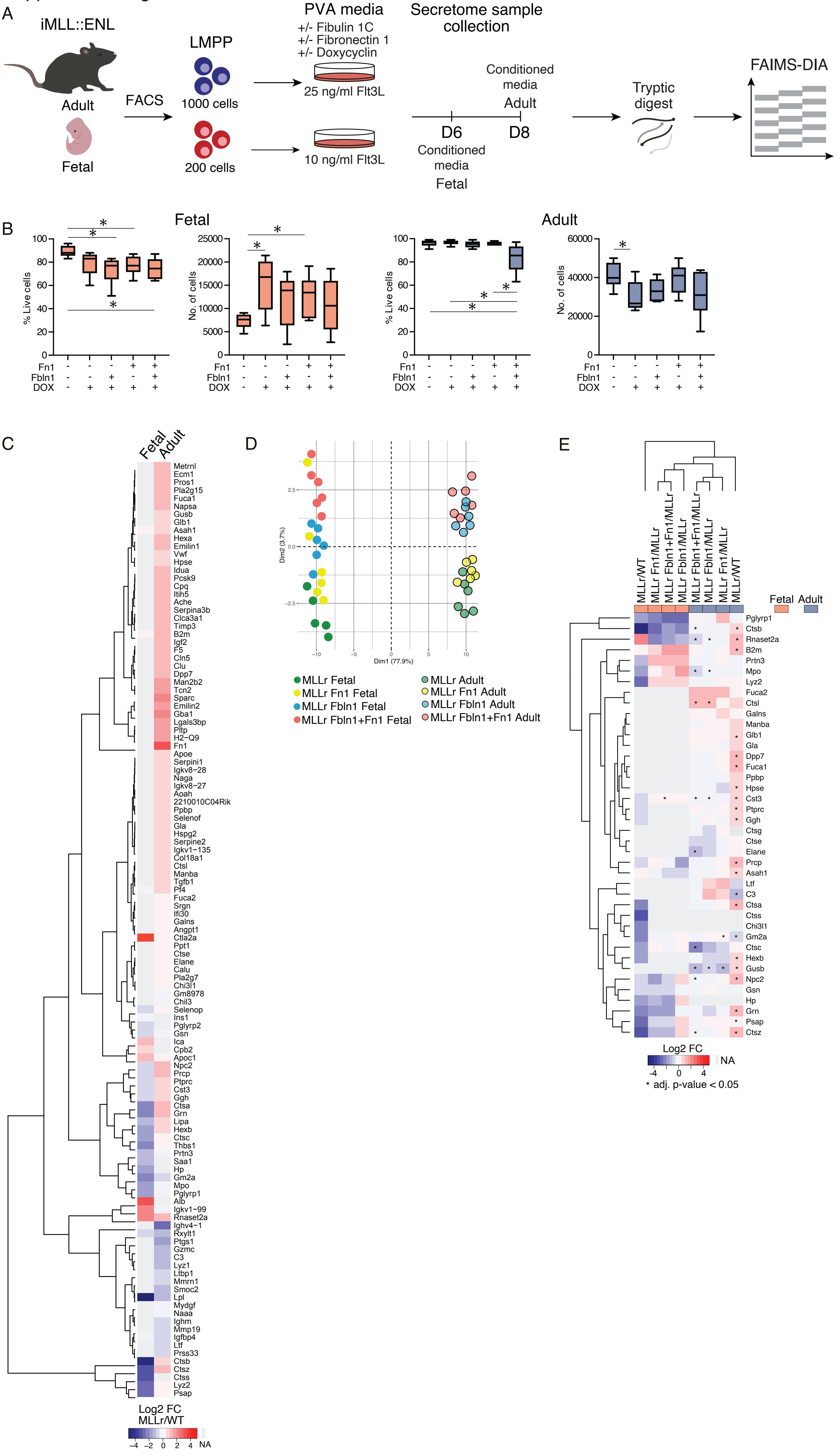

Supplemental Figure 10

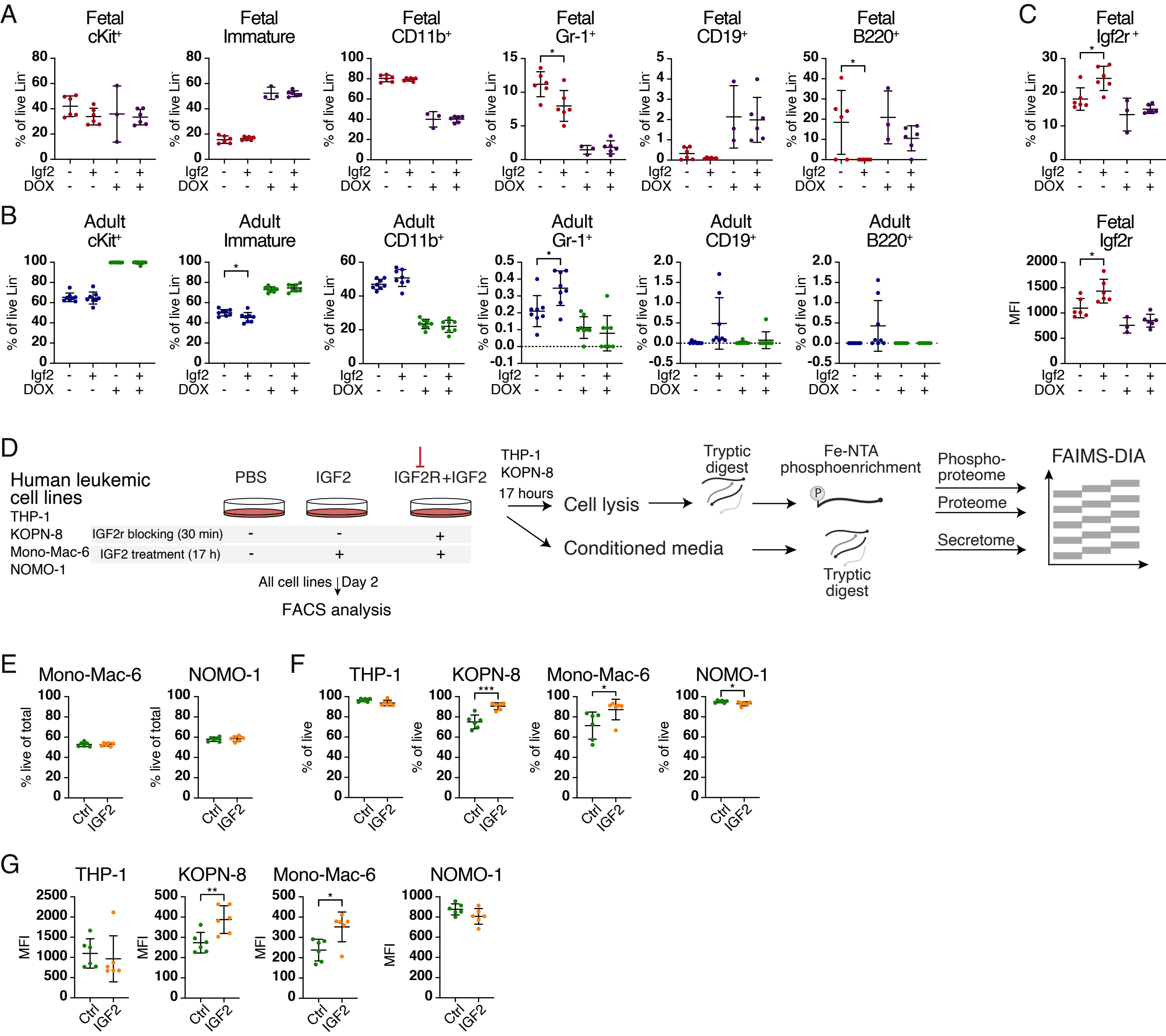

Supplemental Figure 11

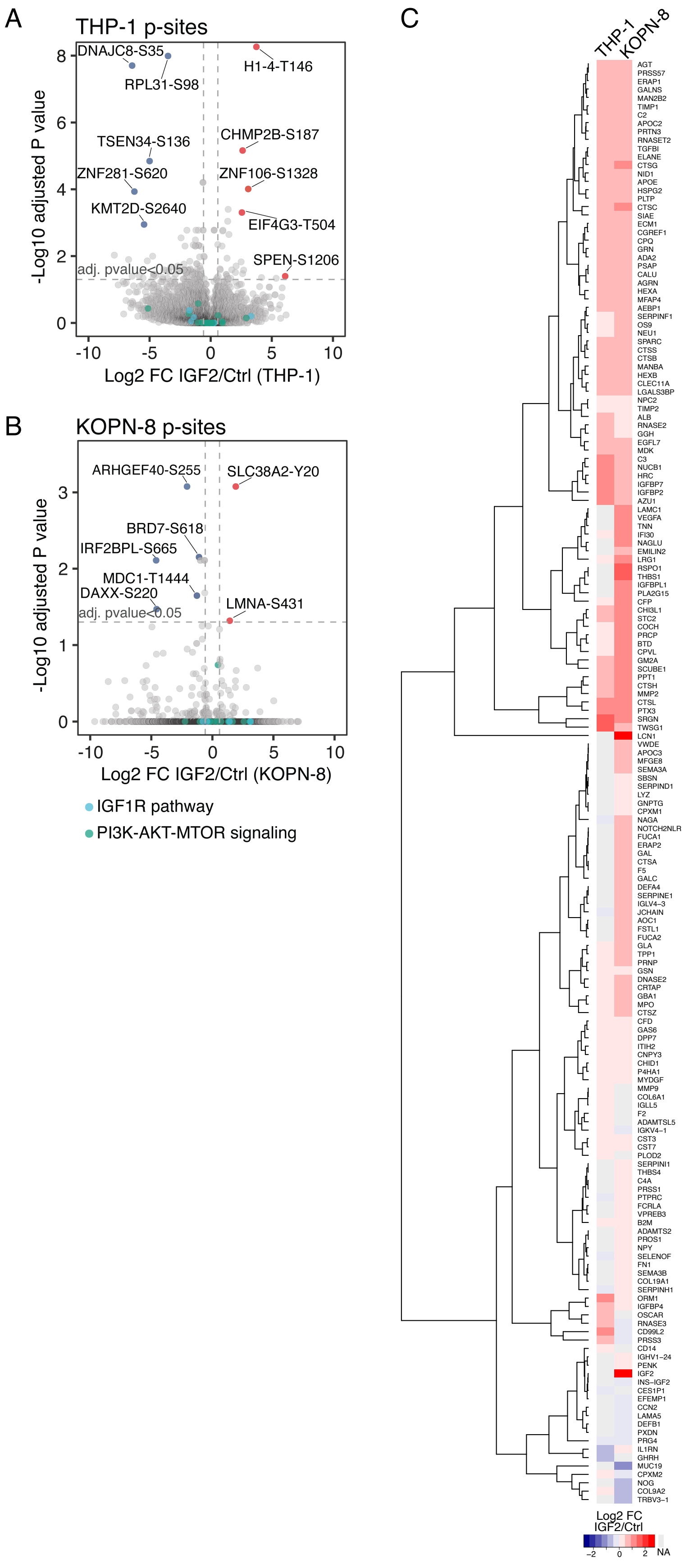

Supplement: Supplementary file 1 — Supplemental Information [file 41375_2024_2235_MOESM1_ESM.pdf]
